# Functions of nitric oxide-mediated post-translational modifications under abiotic stress

**DOI:** 10.3389/fpls.2023.1158184

**Published:** 2023-03-30

**Authors:** Capilla Mata-Pérez, Inmaculada Sánchez-Vicente, Noelia Arteaga, Sara Gómez-Jiménez, Andrea Fuentes-Terrón, Cylia Salima Oulebsir, Mónica Calvo-Polanco, Cecilia Oliver, Óscar Lorenzo

**Affiliations:** Institute for Agrobiotechnology Research (CIALE), Faculty of Biology, University of Salamanca, Salamanca, Spain

**Keywords:** abiotic stress, gasotransmitter, nitroalkylation, nitrosative stress, oxidative stress, reactive oxygen species, reactive nitrogen species, *S*-nitrosylation

## Abstract

Environmental conditions greatly impact plant growth and development. In the current context of both global climate change and land degradation, abiotic stresses usually lead to growth restriction limiting crop production. Plants have evolved to sense and respond to maximize adaptation and survival; therefore, understanding the mechanisms involved in the different converging signaling networks becomes critical for improving plant tolerance. In the last few years, several studies have shown the plant responses against drought and salinity, high and low temperatures, mechanical wounding, heavy metals, hypoxia, UV radiation, or ozone stresses. These threats lead the plant to coordinate a crosstalk among different pathways, highlighting the role of phytohormones and reactive oxygen and nitrogen species (RONS). In particular, plants sense these reactive species through post-translational modification (PTM) of macromolecules such as nucleic acids, proteins, and fatty acids, hence triggering antioxidant responses with molecular implications in the plant welfare. Here, this review compiles the state of the art about how plant systems sense and transduce this crosstalk through PTMs of biological molecules, highlighting the *S*-nitrosylation of protein targets. These molecular mechanisms finally impact at a physiological level facing the abiotic stressful traits that could lead to establishing molecular patterns underlying stress responses and adaptation strategies.

## Background

Plants, as sessile organisms, are regularly challenged by several abiotic stresses involving water availability, temperature fluctuations, UV radiation, or the presence of heavy metals in land. Under normal conditions, aerobic metabolism results in the production of reactive oxygen species (ROS) highlighting superoxide anion ( ${\text{O}}_{2}^{\;.-}$ ), hydrogen peroxide (H_2_O_2_), or hydroxyl radical (**^·^** OH). Likewise, reactive nitrogen species (RNS) are another group of molecules derived from nitric oxide (**^·^** NO) including free radicals like ^·^NO or nitrogen dioxide (**^·^** NO_2_) and non-radicals such as *S*-nitrosothiols (SNO) or peroxynitrite (ONOO^−^) (Halliwell, 2006; del Río, 2015). A NO gasotransmitter has been described to play key regulatory roles in almost all aspects of the plant life cycle as well as responses to (a)biotic stresses with miscellaneous mechanisms that have not been fully understood yet (Freschi, 2013; Yu et al., 2014; Puyaubert and Baudouin, 2014a; Domingos et al., 2015; Trapet et al., 2015; Fancy et al., 2017; Begara-Morales et al., 2018).

**Figure 1:**
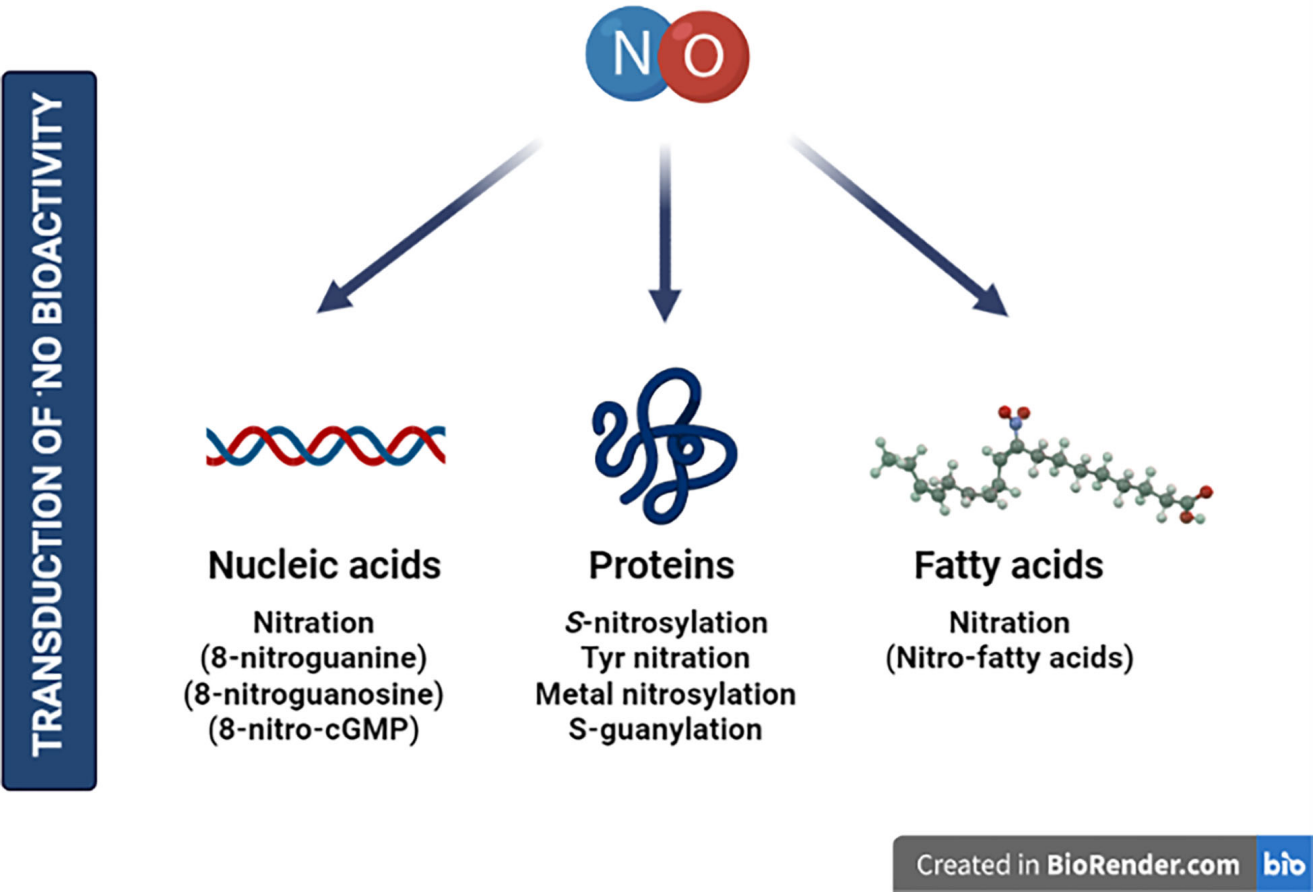
Nitric oxide is considered an essential gasotransmitter for the transduction of bioactivity in plants. Although the main effects described correspond to protein targets, other molecules are susceptible to be modified by NO—including nucleic acids or fatty acids—highlighting molecular points to be explored in future research. Created by BioRender.com.

The exposure to environmental perturbations leads to the accumulation of reactive oxygen and nitrogen species (RONS) in cells. Such increase prompts a reprogramming of their metabolism, especially aimed to neutralize such oxidative or nitrosative stresses (Apel and Hirt, 2004; Radi, 2012). Moreover, abnormal amounts of RONS cause the oxidation of cellular components hampering enzymatic activities or affecting organelle integrity. On the other hand, to counteract the action of these reactive species, enzymatic and molecular antioxidants are produced at the cellular level. These RONS impact plant function through post-translational modifications (PTMs) of macromolecules such as proteins, nucleic acids, or lipids (**Figure 1**). Cysteine (Cys) and methionine (Met) are the most oxidation-susceptible residues within amino acids. The free thiol group (-SH) of Cys can be gradually oxidized to *S*-nitrosothiol (-SNO), sulfenic acid (-SOH), disulfide bridge (-SS-), a covalent attachment of glutathione (*S*-glutathionylation, -SSG), sulfinic acid (-SO_2_H), or sulfonic acid (-SO_3_H) (Spoel and Van Ooijen, 2014). Most of these modifications are readily reversible, with the latter two often described as irreversible. In fact, the reversibility of modifications happening in this intrinsically nucleophilic amino acid provides many different cellular signaling opportunities to regulate plant function.

Furthermore, ^·^NO can interact with ROS, especially with ${\text{O}}_{2}^{\;.-}$ to generate ONOO^−^, which mediates the irreversible nitration of tyrosine residues (NO_2_-Tyr) within proteins, a PTM that alters the protein conformation and mostly provokes loss of function or activity (Radi, 2004). More attention has been given to *S*-nitrosylation, a PTM resulting from the reversible and covalent *S*-linked NO group to the reactive -SH of a Cys residue (**Figure 1**). *S*-nitrosylation is considered one of the key mechanisms for the transduction of ^·^NO bioactivity in plants. This modification modulates protein activities through several mechanisms, including stability, conformation, subcellular localization, biochemical activity, or even protein–protein interactions (Hess et al., 2005; Astier et al., 2011; Astier et al., 2012; Lamotte et al., 2014).

On the other hand, nitration of nucleic acids by RNS also represents one of the key mechanisms mediating the biological activity of NO in all types of organisms (**Figure 1**). Nitration of nucleotides was first described in the early 1990s with the identification of 8-nitroguanine (Yermilov et al., 1995a; Yermilov et al., 1995b; Pinlaor et al., 2003; Ma et al., 2004), 8-nitroguanosine (Niles et al., 2001; Akaike et al., 2003), and 8-nitroguanosine 3’5’-cyclic monophosphate (8-nitro-cGMP) (Sawa et al., 2007) in animal systems. It is noteworthy that 8-nitro-cGMP, being a unique dual signaling molecule, was described to hold powerful redox and electrophilic activities among the identified nitrated guanine derivatives (Sawa et al., 2007). The electrophilic properties of 8-nitro-cGMP can irreversibly modify protein thiols through a novel PTM known as *S*-guanylation (Sawa et al., 2007). It was firstly considered as a marker of nitrosative stress in degenerative diseases, cancer, or other inflammatory conditions (Ohshima et al., 2006); however, recent studies have evidenced biological activities and signaling functions of 8-nitro-cGMP through *S*-guanylation in animal systems (Ihara et al., 2011; Nishida et al., 2016). In plants, there is still insufficient information about the role of nitrated nucleotides on physiology and signaling. However, recent observations have shown the implication of cGMP and 8-nitro-cGMP in *Arabidopsis thaliana* stomatal guard cells opening (Joudoi et al., 2013; Honda et al., 2015).

Finally, fatty acids, especially polyunsaturated fatty acids (PUFAs), are also targeted by RONS. PUFAs are major components of plant membranes and react with ROS through so-called lipid oxidation events, highlighting the peroxidation reactions resulting in the formation of a lipid radical (Vistoli et al., 2013, Alché, 2019). These events occur in plants as a signaling mechanism and after a plethora of stress conditions including high light intensity (Yin et al., 2010). Furthermore, PUFAs, through the activity of lipoxygenase enzymes, are precursors of different signaling molecules, including oxylipins or other oxidized fatty acids, deriving in the production of key phytohormones such as jasmonates (JAs) (Wasternack and Song, 2017). However, a growing body of plant studies is drawing attention to the modification of unsaturated fatty acids by NO and nitrite ( ${\text{NO}}_{2}^{-}$ )-derived RNS leading to nitro-fatty acid (NO_2_-FA) formation. NO_2_-FAs are endogenously present in plant systems mainly in the form of nitro-linolenic (NO_2_-Ln) and nitro-oleic (NO_2_-OA) acids with relevant physiological roles as signaling molecules in (a)biotic stresses, growth, and development (Mata-Pérez et al., 2016c; Arruebarrena Di Palma et al., 2020; Di Fino et al., 2020; Vollár et al., 2020). The mechanisms of action described for NO_2_-FAs involve the ability to act as NO donors, hence modifying proteins through *S*-nitrosylation (Lima et al., 2005; Gorczynski et al., 2007; Faine et al., 2010; Mata-Pérez et al., 2016b) or nitroalkylation (**Figure 1**). The latter is a reversible process based on the strong electrophilicity of the β-carbon adjacent to the nitro group (-NO_2_) and its appetence for soft nucleophiles like Cys or histidine (His) residues (Baker et al., 2007; Geisler and Rudolph, 2012). Thus, nitroalkylation has the capacity to regulate physiological processes in eukaryotes by modulating the activity of transcription factors (TFs) and enzymatic reactions (reviewed in Schopfer et al., 2011; Aranda-Caño et al., 2019). Recently, it has been demonstrated that RONS may be responsible for compromising the stability of this PTM, at least *in vitro*. The oxidation of NO_2_-FA-protein adducts through RONS such as H_2_O_2_ or ONOO^−^ leads to the release of free NO_2_-FAs. Therefore, this oxidation-mediated rupture might act as a mechanism to keep the protein targets sequestered and subsequently released after nitro-oxidative stress (Padilla et al., 2017).

Considering the accumulation of ROS and RNS is a key feature underlying abiotic stressful traits; here, we compile the state of the art about how plants sense, transduce, and integrate the crosstalk between RONS and their impact on plant function and metabolism that could lead us to establish molecular patterns underlying abiotic stress responses and subsequent adaptation strategies in a constantly changing environment.

## Drought and salt stress

**Figure 2:**
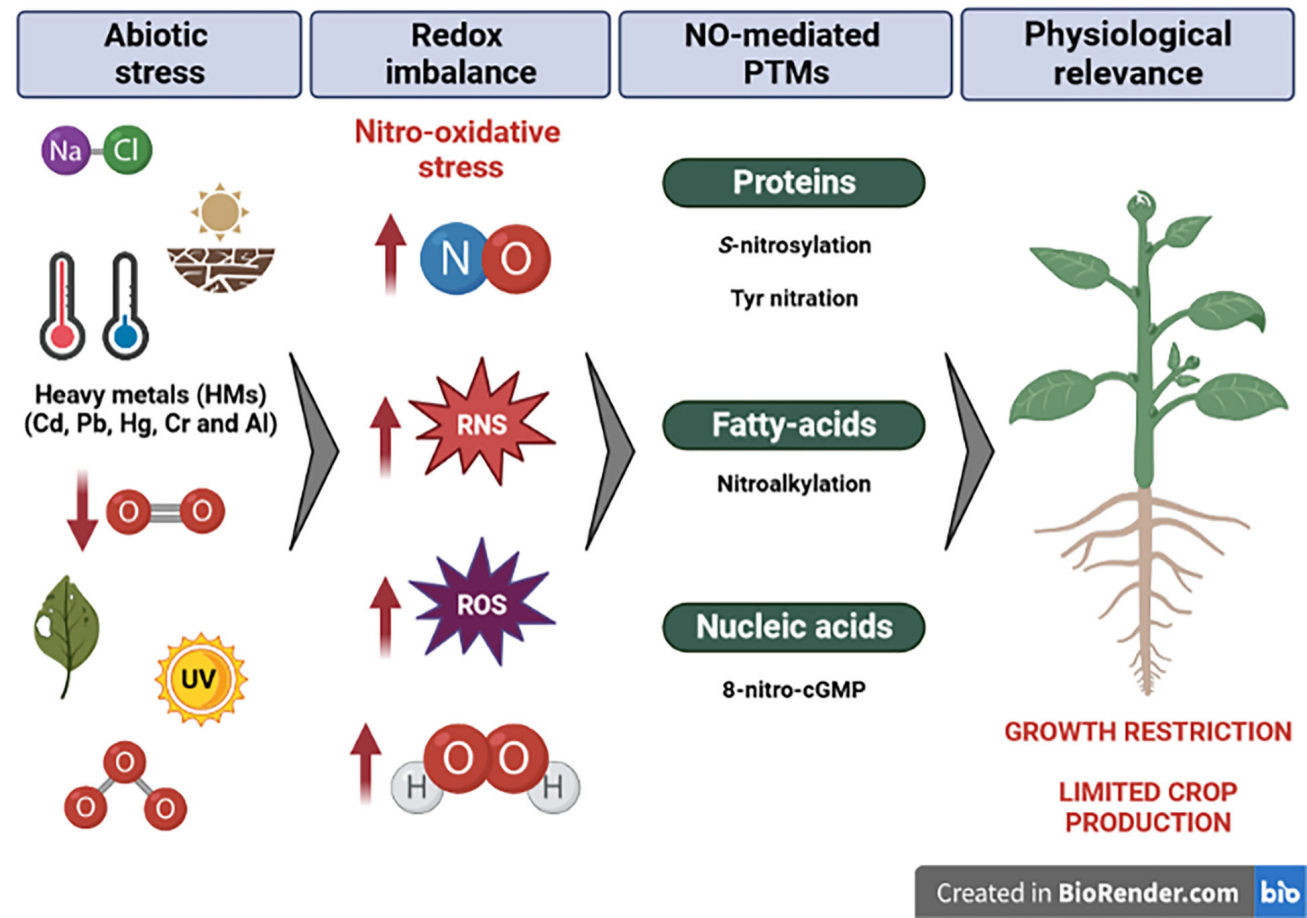
Different abiotic situations promote a redox imbalance linked to nitro-oxidative stress. Plant adaptation implies molecular strategies triggered by NO modifications to alleviate negative effects to readjust their growth and development. Created by BioRender.com.

Plants show a dramatic increase in RONS levels (Bolwell and Wojtaszek, 1997; Navrot et al., 2007; Moreau et al., 2010) that should be controlled to avoid toxic concentrations and to maintain the redox balance during stress. In fact, the alleviation of salt or drought stress through NO is also observed when rat nitric oxide synthase (NOS) is overexpressed in Arabidopsis and rice (Shi et al., 2012; Cai et al., 2015). However, RONS can mainly transmit their activity through PTMs and, among those mediated by RNS, highlight cysteine *S*-nitrosylation or tyrosine nitration of proteins (**Figure 2**).

In Arabidopsis, NO-mediated *S-*nitrosylation has been reported for several TFs involved in abiotic stress responses such as MYB2 or ABI5 (Serpa et al., 2007; Albertos et al., 2015). These TFs participate in abscisic acid (ABA)-mediated regulation under drought stress. MYB2, a member of the MYB TF family, was described to undergo *S*-nitrosylation at Cys53, affecting its DNA binding activity, and indicated to be involved in ABA signaling (Serpa et al., 2007). ABI5 is involved in the repression of germination and seedling establishment (Lopez-Molina and Chua, 2000; Finkelstein et al., 2002). This TF works in the core of ABA signaling together with PYR/PYL/RCAR receptors, PP2C phosphatases, and SnRK2 kinases (Skubacz et al., 2016). ABI5, being a pivotal NO sensor in seeds, is influenced by NO at transcriptional and post-translational levels. Albertos et al. (2015) showed that *S*-nitrosylation of ABI5 at Cys153 facilitates its degradation through the proteasome, therefore leading to both seed germination and seedling growth under favorable conditions, demonstrating an antagonism role between NO and ABA during this process. Moreover, NO also impacts ABA perception through the tyrosine nitration and *S*-nitrosylation of PYR/PYL/RCAR receptors (Castillo et al., 2015). ABA is considered a stress-related hormone; in fact, ABA levels increase during water deficiency (reviewed in Swamy and Smith, 1999). In addition, abiotic stress conditions are known to be accompanied by an increase in RONS, and ABA increase also leads to the accumulation of NO and ROS. This scenario prompts a rise of peroxynitrite (ONOO^−^) content that mediates the nitration of ABA receptors rendering the proteins inactive, targeting them for proteasome degradation and possibly acting as a rapid mechanism to reduce ABA effects. By contrast, *S*-nitrosylation of ABA receptors does not alter their function *in vitro*, suggesting that different NO-related PTMs may act throughout the ABA signaling cascade to attenuate ABA-triggered responses (Castillo et al., 2015). Furthermore, NO regulates the OPEN STOMATA 1 (OST1)/SUCROSE NONFERMENTING 1 (SNF1)-related protein kinase 2.6 (SnRK2.6) by *S-*nitrosylation at Cys137 in cell guards, showing a negative regulation of ABA signaling (Wang et al., 2015). This PTM induces a negative effect on stomata closure during drought stress, observed in cell guards from NO over-accumulating mutants of Arabidopsis (*atgsnor1-2*). Consequently, the *S*-nitrosylation of SnRK2.6 disrupts the ABA-dependent stomata closure (Wang et al., 2015). In the same species, a differential Tyr-nitrated protein profile was observed between wild-type plants and *kea1kea2* (K^+^/H^+^ antiporters) knockout mutants. The latter has closed stomata and shows a higher capacity to resist drought stress compared to the wild-type accession; both results suggest that impaired chloroplast K^+^ could affect the expression of nitrated proteins (Sánchez-McSweeney et al., 2021). Also related to stomatal opening, it has been shown that 8-nitro-cGMP induced stomatal closure in the light, while cGMP did not (Joudoi et al., 2013). The signaling action of 8-nitro-cGMP is mediated by the modulation of Ca^2+^ channels, leading to the activation of SLAC1 (SLOW ANION CHANNEL 1), hence promoting stomatal closure. Furthermore, a metabolite derived from 8-nitro-cGMP, the 8-SH-cGMP, is also involved in the closure of stomata pores (Honda et al., 2015); however, the involvement of *S*-guanylation has yet not been evidenced.

A common and widely described feature underlying salt stress is the increase of the NO and the SNO content (Valderrama et al., 2007; Begara-Morales et al., 2014; Jain et al., 2018). First studies about the involvement of NO-PTMs under salinity were carried out in olive plants where an increase of NO and SNO levels together with a rise of the protein tyrosine nitration profile were described (Valderrama et al., 2007). Sunflower seedlings exposed to NaCl exhibited enhanced tyrosine nitration of cytosolic and oil bodies’ proteins with an enhanced gradient of nitrated proteins from the root tip to the differentiation zone and from the outer layers to the deep-seated cells (David et al., 2015). These results led the authors to think that this is a mechanism to keep oil bodies so that plants can survive longer under salt stress. Jain and Bhatla (2018) demonstrated that the tyrosine nitration of cytosolic peroxidase leads to an increase in its activity after NO exogenous treatment in sunflower seedling cotyledons. However, there is a discrepancy in this regard because the nitration of another peroxidase from the ascorbate–glutathione cycle, the ASCORBATE PEROXIDASE (APX) from *Pisum sativum*, leads to a reduction of its H_2_O_2_ detoxification activity (Begara-Morales et al., 2014). This discrepancy might be explained by the different tissues or plant species explored. Curiously, APX enzyme shows a dual regulation by NO due to the fact that GSNO prompts the enhancement of the activity through *S-*nitrosylation. In fact, under salinity conditions, pea plants show that APX is *S-*nitrosylated; this PTM leads to an increase in its activity (Begara-Morales et al., 2014). Despite the fact that PTM has not been demonstrated, several studies have shown the effect of NO in APX activity under salinity and non-stress conditions. On one hand, several works reveal that APX activity is inhibited after pharmacological treatments with NO donors in different plant species including tobacco and sweet pepper fruits (Clark et al., 2000; González-Gordo et al., 2022). Conversely, an increased APX activity has been demonstrated in sweet potato or soybean plants exposed to NO donors (Keyster et al., 2011; Lin et al., 2011); these results highlight that depending on the plant species and the source of NO applied, the impact on APX bioactivity might vary. On the other hand, the protective effect of NO on different plant species (i.e., barley, soybean, or mustard) exposed to salt stress through the increase of APX activity (Egbichi et al., 2014; Fatma et al., 2016; Yin et al., 2021) and under drought stress in watermelon plants (Hamurcu et al., 2020) has been revealed. Recently, Arabidopsis APX recombinant protein has been shown to be modulated by nitroalkylation, specifically by NO_2_-Ln (Aranda-Caño et al., 2019). This modification leads to a decrease in its enzymatic activity under normal conditions, therefore showing how different NO-related molecules can differentially modulate protein bioactivity. Although this result has been observed under non-stress conditions, nitroalkylation of the peroxiredoxin Tsa1—involved in ROS alleviation—from *Saccharomyces cerevisiae* by NO_2_-OA is abolished under heat stress (Aranda-Caño et al., 2022). The authors considered that nitroalkylation of Tsa1, in the absence of stress, is a way to keep the enzyme sequestered and inactive, while, upon stress, where a rise of RONS takes place, the enzyme can be released and trigger its antioxidant properties. We cannot rule out that something similar might be happening to APX where, upon drought or salinity stress, nitroalkylation can release APX, leading to H_2_O_2_ detoxification.

Similarly in pea plants, Camejo et al. (2013) demonstrated that salinity stress induces the *S-*nitrosylation of PEROXIREDOXIN IIF (PrxIIF) in the mitochondria, which reduces its peroxidase activity, due to a conformational change in the protein structure, acquiring a transnitrosylase activity and subsequently broadening the effects of protein *S*-nitrosylation (Camejo et al., 2013; Camejo et al., 2015).

Finally, there are other pieces of evidence about NO implication during salt stress alleviation that are described below. Polyamine (PA) biosynthesis is activated and induces the accumulation of H_2_O_2_ and several antioxidant activities during salinity (Tanou et al., 2014). In citrus plants, putrescine or spermidine led to the suppression of protein carbonylation and tyrosine nitration whereas protein *S*-nitrosylation was elicited by these molecules. Furthermore, a proteomic approach on citrus plants concomitantly exposed to PAs and NaCl showed the *S*-nitrosylation of catalase enzyme, which was accompanied by an induction of its enzymatic activity, suggesting an interplay between protein *S*-nitrosylation and protein carbonylation (Tanou et al., 2014). The positive role of NO in salt stress alleviation is also evident in tomato seedlings, showing an increase in *S*-nitrosylated proteins under NaCl treatment (Wang et al., 2022; Wei et al., 2022). Some of them are involved in MAPK signaling showing a downregulation in S-adenosyl-L-methionine (SAM) proteins like SAM1 and SAM3 (precursor of ethylene biosynthesis), SnRK, and PP2C, and upregulation of *MAPK*, *MAPKK*, and *MAPKK5* at the transcriptional level when plants grew under salt stress plus GSNO, protecting tomato seedlings from this stress (Wei et al., 2022). Another evidence about the role of NO during salt stress alleviation is the increase of the tomato MONODEHYDROASCORBATE REDUCTASE (MDHAR) activity through *S*-nitrosylation after exposure to salt stress, due to the important role of this enzyme during the ascorbic acid regeneration during ROS detoxification (Qi et al., 2020).

## High and low-temperature stress

Heat and cold stresses promote changes that affect plants at the molecular level leading to plant dysfunction (Sánchez-Vicente and Lorenzo, 2021). The tolerance of plants to non-optimal temperatures seems to be linked to the redox regulatory system, which integrates information from metabolism through thiol-containing proteins to modulate cell status and minimize cellular damage (Dietz, 2008).

Heat stress (HS) deeply impacts plant development, including seed yield losses and crop quality (Sehgal et al., 2018), seed germination in Arabidopsis (Toh et al., 2008), leaf senescence in bent grass (Rossi et al., 2017), and photosynthetic damage (Wang et al., 2018). HS causes an increment in the oxidation level in both the nucleus and cytosol, which could be related to both genetic and epigenetic adaptive reprogramming (Babbar et al., 2021). The role of NO has been widely described under high temperatures (Lee et al., 2008), having shown that mutants impaired in NO homeostasis display abnormal growth and development, and the seed production is compromised under different temperatures (Sánchez-Vicente and Lorenzo, 2021). In addition, both deficient and SNO/NO over-accumulators are affected in the response to HS and thermotolerance capacities (Lee et al., 2008; Xuan et al., 2010). NO mechanisms during plant acclimation to HS include activation of cellular responses through the *S*-nitrosylation of the trihelix TF GT-1, which promotes its binding to *HEAT SHOCK TRANSCRIPTION FACTOR A2* (*HsfA2*) promoter (He et al., 2022). During germination, Cys *S*-nitrosylation has been described as an important PTM, where high temperature and the photoreceptor phytochrome B (phyB) modulate antagonistically LONG HYPOCOTYL IN FAR-RED (HFR1)-SNO to coordinate seed thermotolerance (Ying et al., 2022).

Tyr nitration is also a PTM closely related to HS response. Large-scale analyses have shown great changes in the nitroproteome of *Helianthus annuus* seedlings exposed to HS, with the FERREDOXIN–NADP REDUCTASE (FNR) (Chaki et al., 2011) and CARBONIC ANHYDRASE (CA) being two of these proteins modulated by Tyr nitration (Chaki et al., 2013). These modifications inhibit its activity, finally impacting the photosynthesis process. Also related to nitration but of fatty acids (NO_2_-FAs), it has been shown that exogenous treatments with NO_2_-Ln induce the expression of genes involved in the HS response and the acquisition of thermotolerance, highlighting heat shock proteins (HSPs) and heat shock TFs (HSFs) (Mata-Pérez et al., 2016a).

Like HS, cold stress constitutes a harmful situation as an important yield-limiting factor. Under low temperatures, plants suffer changes at the biochemical and physiological levels, leading to transcriptional modifications, cellular dysfunction, changes in the membrane composition and fluidity, metabolic imbalance, and changes in enzyme activity (Manasa et al., 2022). Plant acclimation and adaptation to cold conditions implies a profound reorganization of transcriptome, metabolome, and proteome. In *Brassica juncea*, low-temperature stress led to important differences in the nitrosoproteome, which can be related to cold stress-induced photosynthetic inhibition (Abat and Deswal, 2009). In this context, previous research showed modulation of cold-responsive proteins by *S*-nitrosylation as an important cue to attenuate stress, focusing on the regulation of nuclear trafficking to control cellular metabolism and redox status (Sehrawat et al., 2019). Furthermore, it has been recently described a rise in the *S*-nitrosylation level by brassinosteroids under low-temperature conditions in mini-Chinese cabbage seedlings to ameliorate plant damage (Gao et al., 2022). Furthermore, NO acts downstream of H_2_O_2_ and cooperates with JAs in freeze tolerance (Liu et al., 2019). In *Pisum sativum*, an increase in both *S*-nitrosylation and Tyr nitration has been detected during cold stress situations (Corpas et al., 2008; Airaki et al., 2012), highlighting the central role of PTMs during its perception and response. Both *S*-nitrosylation and Tyr nitration play a role during cold stress responses, controlling photosynthetic, metabolic, defense, and signaling-related proteins that lead to adaptive responses, highlighting HSPs, GST, DHAR, RuBisCO, GAPDH, and SAHH1/HOG1 (Sehrawat et al., 2013; Puyaubert et al., 2014b). These results point to a possible crosstalk between different redox PTMs during stress adaptation, involving genetic and metabolic reprogramming. Overall, results derived from these studies show how PTMs, and especially those triggered by RONS, are crucial for the perception and response to stressful situations related to drastic temperature alterations.

## Mechanical wounding

From all the stress a plant faces, injury is one of the most common; it can be caused by (a)biotic factors such as rain, wind, herbivores, and insects. It offers an easy way of entry for opportunistic pathogens through the open wounds. The defenses activated by mechanical injury (wounding) are like the ones activated by herbivores and insects (Reymond et al., 2000; Arimura et al., 2005; Rehrig et al., 2014); thus, they can mimic a biotic stress-like trait.

In response to pathogens and wounding, the emerging signaling molecules in plant immunity activation are ROS (Mittler et al., 2011; Suzuki and Mittler, 2012). When an injury occurs, the immediate region is the first to react. Among the changes that take place in the plant are alterations in the Ca^2+^ concentration in the plasma, the synthesis of secondary messengers, RONS, and a sharp increase in the levels of fight hormones such as JA and ABA (Mostafa et al., 2022). A cell-to-cell process allows Ca^2+^ and ROS to go through the xylem, parenchyma, and phloem tissues to reach the undamaged areas of the plant (Farmer and Ryan, 1992). A feedback loop between NO and ROS has been shown to exist, and ROS/NO balance is a crucial factor in determining how cells respond to abiotic stress caused by antioxidant defenses and ROS generation. Direct NO-dependent protein regulation is the subject of another area of study, particularly through the main NO-PTMs such as *S*-nitrosylation and protein nitration (**Figure 2**) (Neill et al., 2003; Neill et al., 2007; Astier et al., 2012). The NO_2_-Tyr levels in sunflower hypocotyl cells have been shown to rise following mechanical damage (Chaki et al., 2010). The authors suggested that the damage resulted in a buildup of GSNO under oxidative stress. The process of tyrosine nitration brought on by ONOO^−^ production may be mediated by *S*-nitrosothiols (SNOs). This is most likely caused by the fact that in the presence of oxygen (O_2_), GSNO degrades to the subsequent radicals, glutathione (GS) and ONOO^−^, which are responsible for the observed rise in protein tyrosine nitration levels. In conclusion, sunflower seedlings are subjected to nitrosative stress, and SNO may act as a unique injury signal in plants (Chaki et al., 2010).

Extrafascicular phloem (EFP) is a defensive structure against herbivorous animals developed by cucurbits. Mechanical leaf wounding induces a systemic wound response through the tight regulation of RONS signaling. Quickly after the injury, the activities of key antioxidant enzymes like dehydroascorbate reductase (DHAR), glutathione reductase (GR), and ascorbate peroxidase (APX) decreased together with ascorbate and glutathione content. Opposite behavior on NO-PTMs was observed with a decrease in protein *S*-nitrosylation and an increase in protein tyrosine nitration over time. Collectively, the accumulation of ROS within the EFP leads to a change in RONS composition from NO to its more nitrating species including ONOO^−^ and NO_2_ curving the stress dynamics and the redox status after mechanical wounding (Gaupels et al., 2016). Another study performed in sea rockets (*Cakile maritima* L.) pinched with striped-tip forceps showed a differential modulation in the damaged hypocotyls and unwounded organs. Wounded hypocotyls exhibited an active RONS metabolism with increased protein nitration in green cotyledons causing long-distance signals that could elicit responses in unwounded tissues. These data, therefore, confirm the existence of local and long-distance responses that counteract negative effects and provide appropriate responses, enabling the wounded seedlings to survive (Houmani et al., 2018).

Other studies in tomato plants have shown that NO may have a role in downregulating the expression of wound-inducible genes during pathogenesis (Orozco-Cárdenas and Ryan, 2002). To activate wound signaling, tomato plants respond to injury and elicitors by accumulating high amounts of H_2_O_2_, and it has been suggested that NO may function as an antioxidant, preventing ROS damage to plant cells and tissues (Lin et al., 2011). Closely related, mechanical wounding enhances freezing tolerance in untreated systemic leaves of wheat plants through the accumulation of NO and H_2_O_2_ and further modifications in the photosystem and antioxidant system (Si et al., 2017). Also, after injuring potato leaflets, a NO burst takes place, which is required to start the healing process (París et al., 2007). Overall, RONS are key molecules in mediating the response of the plant to mechanical injuries.

## Heavy metals

The definition of heavy metals (HMs) was established by Hawkers in 1997 as the group of metals and metalloids with an atomic density greater than 4 g/cm^3^, or five times or more than that of water (Hawkes, 1997). However, their chemical properties make HMs both indispensable and toxic, even at low concentrations, for plants (Duruibe et al., 2007; Zitka et al., 2013). HMs are classified as essentials and nonessentials. Essential HMs, like Cu, Fe, Mn, Co, Zn, or Ni, are required for fundamental biological processes as they serve, for instance, as enzyme cofactors, whereas nonessential HMs, such as Cd, Pb, Hg, Cr, and Al, are not required by plants for their metabolic processes (Zitka et al., 2013; Raychaudhuri et al., 2021).

HMs have been considered major environmental pollutants altering plant metabolism, growth, and biomass production (Nagajyoti et al., 2010) and essential HMs are even toxic at excessive concentrations (Rascio and Navari-Izzo, 2011; Hossain et al., 2012). Those elements negatively affect plant molecular physiology and biochemistry by generating several stresses including oxidative and nitrosative ones (Hoque et al., 2021). The major risk of HM poisoning is the enhanced production of ROS (**Figure 2**), as they usually interfere with natural ROS metabolism and homeostasis, exposing cells to oxidative stress (Nagajyoti et al., 2010; Zitka et al., 2013; Zandi and Schnug, 2022). With this aim, numerous studies have demonstrated a relationship between ROS-scavenging systems and HMs in different plant species such as *Colobanthus quitensis* (Contreras et al., 2018), *Vaccinium myrtillus* L. (Kandziora-Ciupa et al., 2013), and *Pteris vittate*, which is considered an HM-resistant plant (Srivastava et al., 2005). Furthermore, RNS metabolism has also been defined as a mechanism of response against HMs, where NO has shown a protective effect under HMs throughout protein *S*-nitrosylation (**Figure 2**), as previously reviewed (Gill et al., 2013; Romero-Puertas et al., 2013; Saxena and Shekhawat, 2013). There is also an overproduction of ONOO^−^ in Arabidopsis root peroxisomes (Corpas and Barroso, 2014) and an alteration of the protein tyrosine nitration profile under HMs (Feigl et al., 2016; Kolbert et al., 2018) or its derivatives such as arsenate (Leterrier et al., 2012) or indium (Zhao et al., 2022). The response to HMs is usually linked to RONS production and antioxidant system modulation. For example, the presence of Cd in wheat has shown that NO can significantly increase plant resistance by reducing ROS accumulation as well as increasing the antioxidant defense system and nutrient assimilation (Kaya et al., 2020), therefore reinforcing the role of NO in alleviating abiotic stress. In the case of Zn, an interplay between H_2_O_2_ and *S*-nitrosothiol signaling has been described in Arabidopsis plants, throughout the inactivation of GSNO reductase by Zn-induced H_2_O_2_ (Kolbert et al., 2019).

Furthermore, the protective role of NO in Cd toxicity has been related to the reduction of lipid peroxidation and the inhibition of H_2_O_2_ accumulation at a molecular level in barley, where SNP application significantly reduced Cd growth inhibitory effects by the improvement in chlorophyll content, regulating the activity of antioxidant enzymes and causing a decrease in lipid peroxidation and MDA content (Alp et al., 2022). In addition, as NO is tightly associated with phytohormone signaling, some studies have been aimed to understand how the interaction between NO and phytohormones is altered during HM stress and tolerance (Asgher et al., 2017; Demecsová and Tamás, 2019; Pande et al., 2022). In this context, NO has been shown to reduce Al accumulation in the root apices of wheat regulating the hormonal equilibrium of gibberellins and auxin, enhancing plant tolerance to Al stress (He et al., 2012). There is a complex NO-auxin crosstalk involved in HM stress, where long-term Cu^2+^ exposure causes an increase in NO production and a repression of auxin by inhibiting PIN1-mediated auxin transport-dependent gene expression in Arabidopsis root tips (Kolbert et al., 2012). In the case of *Medicago truncatula*, NO supplementation improves Cd stress tolerance by reducing the activity of IAA oxidase to maintain auxin equilibrium (Xu et al., 2010). Something similar happens in tomato roots during Cd stress, where the addition of IAA with Cd upregulates components of the AsA-GSH cycle for balancing ROS (Khan et al., 2019).

Thus, a clear relationship between RONS metabolism as a defense mechanism against HMs toxicity has been reported. Several studies carried out in plants have demonstrated the impact of potentially toxic HMs on the homeostasis of ROS-scavenging systems and the protective effects of NO under HM stress.

## Hypoxia

Plants, as aerobic organisms, rely on oxygen for their respiration and mitochondrial energy generation. However, different developmental and environmental conditions can lead to decreased O_2_ levels (Weits et al., 2021) such as soil waterlogging and submergence (caused by excessive rain) or pathogen infection. As a result, plants face hypoxia stress when partial O_2_ deficiency (usually between 1% and 5% O_2_) limits aerobic respiration, while anoxia takes place in total O_2_ depletion (Sasidharan et al., 2017). This situation leads to stress-related effects such as lack of energy, saturated electron transfer chain, or high levels of reducing equivalents, thus constraining growth and crop productivity (reviewed in Jethva et al., 2022).

To survive during environmentally low O_2_ conditions, plants have developed several morphological and physiological mechanisms of adaptation (Kende et al., 1998; Drew et al., 2000). O_2_ deficiency significantly decreases ATP content due to restrictions on aerobic metabolism (Bailey-Serres and Voesenek, 2008). As a result, plants need to re-route their energy metabolism to guarantee survival, leading to cell acidification and the accumulation of RONS like NO, which potentially lead to cell damage when their production exceeds threshold levels (Hebelstrup and Møller, 2015; Turkan, 2018). However, under moderate concentrations, these factors act as key signaling molecules involved in stress adaptation responses through transcriptional regulation, enzyme activity, and secondary messengers that help maintain cellular homeostasis (Gaupels et al., 2011; Mengel et al., 2013; Lamotte et al., 2015).

Although RONS are involved in many processes aimed to get a fine-tuned response to hypoxia, we have focused on key PTMs triggered by NO (Manrique-Gil et al., 2021) (**Figure 2**). *S*-nitrosylated proteins potentially implicated in flooding signaling and adaptation include ACONITASE (Gupta et al., 2012), CYTOCHROME C OXIDASE (COX) (Millar and Day, 1996), PHYTOGLOBIN 1 (PGB1) (Perazzolli et al., 2004), membrane-bound NADPH oxidases (RBOHs) (Yun et al., 2011), and GSNO REDUCTASE 1 (GSNOR1) (Zhan et al., 2018).

Under hypoxia, inhibition of ACONITASE by *S*-nitrosylation leads to the accumulation of several metabolic intermediates such as citrate, which induces *ALTERNATIVE OXIDASE 1A* (*AOX1A*) expression and increases its activity in Arabidopsis (Gupta et al., 2012). NO is an inducer for AOX (Royo et al., 2015), but a NO burst may inhibit COX activity (Millar and Day, 1996; Brown, 2001). This altered bioactivity of AOX and COX suggests that NO plays a role in plant mitochondrial respiration under hypoxic conditions (reviewed in Sasidharan et al., 2018). In this context, *AOX* induction can promote anaerobic ATP synthesis, which increases energy efficiency as an adaptive response to hypoxia (Millar and Day, 1996; Vishwakarma et al., 2018). This suggests that AOX overexpression in crops could be a target in breeding programs aimed at flooding and waterlogging tolerance. In addition, redox mechanisms are also involved in the post-translational regulation of AOX activity by the formation of a thiohemiacetal at CysI with 2-oxo acids (Gupta et al., 2009; Selinski et al., 2017; Selinski et al., 2018). Under normoxia, AOX limits uncontrolled ROS production by preventing over-reduction of the electron transport chain (reviewed in Selinski et al., 2018). However, under hypoxic conditions, AOX limits superoxide generation and increases NO production, thus preventing nitro-oxidative stress during re-oxygenation (Vishwakarma et al., 2018; Jayawardhane et al., 2020). In 2018, Vishwakarma and colleagues concluded that *AOX* has a distinct role depending on the O_2_ availability since AOX can produce NO under hypoxia whereas it scavenges NO in normoxic conditions.

Another crucial strategy of adaptation under hypoxia relies on the phytoglobin/NO cycle, in which phytoglobins (PGBs) modulate NAD(P)^+^ and nitrate regeneration under hypoxia, which, in turn, fuels glycolysis and ATP production (Perazzolli et al., 2004; Igamberdiev et al., 2005; Gupta and Igamberdiev, 2016). This cycle helps to retain the energy status of the plant through NO scavenging by PGB1 *via S*-nitrosylation (Perazzolli et al., 2004; Rubio et al., 2019), metal nitrosylation (Perazzolli et al., 2004), and Tyr nitration (Sainz et al., 2015).

RBOHs are key proteins involved in plant responses to hypoxia by modulating ROS signaling (Rajhi et al., 2011; Pucciariello et al., 2012; Yeung et al., 2018). In several species, RBOH-mediated ROS accumulation in the root cortical cells leads to programmed cell death and thus to the generation of flooding-induced aerenchyma tissue (Rajhi et al., 2011; Yamauchi et al., 2014; Yamauchi et al., 2017). The impact of RBOH in ROS production is tightly regulated *via* several PTMs, including NO-mediated *S*-nitrosylation (Yun et al., 2011). Yun et al. studied the NO and reactive oxygen intermediaries’ (ROIs’) involvement in hypersensitive response (HR) during microbial infection. Their results show that *S*-nitrosylation of AtRBOHD at Cys 890 reduces its activity, limiting the cell death caused by stress-induced oxidative bursts.

NO signaling is also linked to autophagy in the hypoxic response in Arabidopsis since *S*-nitrosylation of GSNOR1 at Cys 10 induces conformational changes that lead to its selective degradation in the autophagosome (Zhan et al., 2018). NO can also react with ${\text{O}}_{2}^{-}$ , producing ONOO^−^ and reducing H_2_O_2_ generation, which might avoid cell death (Chen et al., 2009).

NO-related PTMs are thought to be involved in hyponastic leaf movement (Hebelstrup et al., 2012) and aerenchyma generation (Wany et al., 2017), both ethylene-mediated flood-adaptive traits (reviewed in Sasidharan et al., 2021). Regarding aerenchyma formation, Wany, Kumari, and Gupta demonstrated that not only ethylene, but also hypoxia-induced NO plays an important role in root cortical cell death. It has been reported that NO modulates ROS production, lipid peroxidation, and tyrosine nitration, but its mechanistic role in aerenchyma generation is still unknown. However, the observation of a nitrosylated 63-kDa protein suggests that ONOO^−^ and changes in protein activity may be involved. NO is also important for these ethylene-mediated adaptive responses since it can activate ethylene biosynthesis, potentially by *S*-nitrosylation of key enzymes such as ACC synthase and oxidase (Li et al., 2016).

## UV radiation

Plants and algae produce organic matter from CO_2_ and water using light energy in a process called photosynthesis, which takes place in the chloroplasts. However, light is one of the major stress factors resulting in ROS generation in chloroplasts that produces photo-oxidative damage, the inhibition of photosynthesis, and cell death (Li et al., 2009; Shi et al., 2022). Light information is perceived by both chloroplasts, where photosynthesis takes place, and photoreceptors, which act in response to light initiating a signaling process (Li et al., 2009).

UV-B (280-315 nm) is a minor component of sunlight that is receiving special interest from researchers as it is increasing because of stratospheric ozone reduction (Caldwell et al., 2003). As happens in several stress responses, hormones act downstream of the UV-B signaling pathway (Vanhaelewyn et al., 2016). In response to UV-B, the production of ROS increases, with multiple sources responsible for this production (Mackerness et al., 2001). This increase in ROS levels leads to the production of hormones such as salicylic acid, ethylene, and JA, which play a role in response to multiple stress conditions (Reymond and Farmer, 1998). UV resistance locus8 (*UVR8*) is a photoreceptor (Kliebenstein et al., 2002) that localizes as a dimer in the cytoplasm and, in the presence of UV-B, accumulates as monomers in the nucleus where it interacts with the multifunctional E3 ubiquitin ligase CONSTITUTIVELY PHOTOMORPHOGENIC (COP1), abolishing its function and, as a consequence, causing the accumulation of its targets such as *ELONGATED HYPOCOTYL5* [*HY5*, a basic leucine zipper (bZIP) TF] and *HY5 HOMOLOG* (*HYH*) (Kaiserli and Jenkins, 2007; Brown and Jenkins, 2008; Favory et al., 2009). Both HY5 and HYH play an antagonistic role to PHYTOCHROME INTERACTING FACTOR1 (PIF1) and PIF3 in regulating cell death and photooxidative response (Chen et al., 2013; Vanhaelewyn et al., 2016). In parallel, NO levels increase in response to UV-B in plants (Mackerness et al., 2001; Zhang et al., 2003) and it acts as an important factor in protecting plants against UV-B effects. It has been reported that when plants with a decrease of endogenous NO are exposed to UV-B radiation, damaged symptoms are enhanced (Cassia et al., 2019). The perception of UV-B by UVR8 leads to an increase in these NO levels regulating the stomatal closure, protecting the microtubules organization, scavenging ROS, and upregulating *HY5* (Krasylenko et al., 2012; Tossi et al., 2014; Li et al., 2022). Moreover, UV-B light response includes a crosstalk among H_2_O_2_ (produced by AtRBOHD and AtRBOHF), NO, and UVR8 (He et al., 2013; Wu et al., 2016). However, the precise role of NO in this relationship among them has not been studied yet, opening the possibility of a post-translational regulation of some of the key proteins in UV-B response by NO.

## Ozone

Tropospheric ozone (O_3_) is considered a major phytotoxic air pollutant that causes detrimental effects in ecosystems and agricultural systems worldwide (Ainsworth et al., 2012; Tai et al., 2021; Wu et al., 2022), and it is formed through the action of light-driven chemical reactions involving nitrogen oxides (NOx) and volatile organic compounds. Ozone is a powerful oxidizing agent that accesses plants *via* stomata and breaks into ROS in the apoplast (Ainsworth, 2017; Waszczak et al., 2018). High levels of O_3_ in plants induce decreases in photosynthesis and stomatal conductance rates, photosynthetic proteins, and pigments (Brosché et al., 2010; Kontunen-Soppela et al., 2010; Ainsworth, 2017; Nanni et al., 2022), chloroplast development (Nagatoshi et al., 2016), and cell death (Overmyer et al., 2005; Horak et al., 2016; Sierla et al., 2018). Transcriptional reprogramming in O_3_-affected plants has been identified in several species (revised in Morales et al., 2021), and the TF families studied include the *Ethylene Response Factors* (*ERF*), *TGA*, *WRKY* (Xu et al., 2015; Hoang et al., 2017; Morales et al., 2021), and *cysteine-rich receptor-like kinases* (*CRKs*) encoding regulators of hormone signaling (Wrzaczek et al., 2010). Furthermore, the mechanisms of plant responses to O_3_ have been linked to the function of the mitogen-activated protein kinase 12 (MAPK12) and the transcription reprogramming of the plants. Among them, the TF GOLDEN2-LIKE (GLK1 and GLK2) was related to the O_3_ tolerance of plants through the regulation of stomatal movement (Fiscus et al., 2005; Puckette et al., 2008; Brosché et al., 2010; Nagatoshi et al., 2016; Mills et al., 2018) and the WKRY and MYB families of TFs with the control of anthocyanin and proanthocyanidin biosynthesis (Duan et al., 2018; Zhang et al., 2022). Moreover, it has been shown that O_3_ stimulates the production of NO (Ederli et al., 2006; Ahlfors et al., 2009; Kabange et al., 2022), probably by the action of the nitrate reductase (NR) (Xu et al., 2012). NO induced by ozone will contribute to the generation of ROS and different signaling pathways in plants (Hasan et al., 2021; Mukherjee, 2022), possibly altering NO-ROS balancing or the plant hormonal homeostasis (Ahlfors et al., 2009). The mechanisms of NO regulation induced by O_3_ were studied through the proteome analysis in poplar leaves. High levels of O_3_ induced changes in total nitrite and *S*-nitrosothiol contents and affected the *S*-nitrosylated status of proteins (**Figure 2**) (Vanzo et al., 2014). Hence, key proteins related to the phenylpropanoid pathway (PAL2), photosynthetic activity (Chlorophyll a/b binding proteins), and cell wall composition (alpha-N-arabinofuranosidase) were significantly de-nitrosylated after ozone exposure, while others were putative targets of *S*-nitrosylation, such as the Ribulose-phosphate 3-epimerase, the Peroxiredoxin 5, and the Tubulin alpha-chain (revised in Vanzo et al., 2014).

In conclusion, the mechanisms of plant responses to high O_3_ are complex and far from being completely understood. The exposure of plants to O_3_ will promote different molecular and physiological responses related to ROS and NO that will vary according to the plant sensitivity to this pollutant, with broad implications on plant defense mechanisms that will be critical for their adaptation to a constantly changing environment.

## Conclusions and future perspectives

Plant welfare and crop yield are continuously influenced by environmental factors, pests, or nutrient availability in the soil. Abiotic stresses hamper plant fitness by impacting the morphology, biochemistry, and physiology, which are tightly connected to the growth and yield of the plant. Nitro-oxidative stress is a common feature underlying abiotic stresses. The ROS and RNS produced can transduce their bioactivity through the post-translational modification of biomolecules, therefore modulating the molecular mechanisms involved in the redox control of plant processes. Although many nitrated and *S*-nitrosylated proteins have been identified, new protein modifications mediated by nitro-fatty acids or nucleic acids—including nitroalkylation or *S*-guanylation*—*have been scarcely explored during abiotic stress. In this context, more research is needed to better comprehend the biological implications of these NO-modified biomolecules into the redox regulation of abiotic stresses. The data provided here expand our understanding of how NO and NO-related molecules, through the post-translational modification of biomolecules, can modulate the redox fitness, therefore providing the biological framework for future research to improve plant tolerance to abiotic stress.

## Author contributions

All authors contributed equally to the conceptualization, writing of the original draft, review and editing. OL is responsible for supervision and funding acquisition.

## References

[B1] Abat J. K. Deswal R. (2009). Differential modulation of s-nitrosoproteome of brassica juncea by low temperature: change in s-nitrosylation of rubisco is responsible for the inactivation of its carboxylase activity. Proteomics 9, 4368–4380. doi: 10.1002/pmic.200800985 19655309

[B2] Ahlfors R. Brosché M. Kollist H. Kangasjärvi J. (2009). Nitric oxide modulates ozone-induced cell death, hormone biosynthesis and gene expression in arabidopsis thaliana. Plant J. 58, 1–12. doi: 10.1111/j.1365-313X.2008.03756.x 19054359

[B3] Ainsworth E. A. (2017). Understanding and improving global crop response to ozone pollution. Plant J. 90, 886–897. doi: 10.1111/tpj.13298 27739639

[B4] Ainsworth E. A. Yendrek C. R. Sitch S. Collins W. J. Emberson L. D. (2012). The effects of tropospheric ozone on net primary productivity and implications for climate change. Annu. Rev. Plant Biol. 63, 637–661. doi: 10.1146/annurev-arplant-042110-103829 22404461

[B5] Airaki M. Leterrier M. Mateos R. M. Valderrama R. Chaki M. Barroso J. B.. (2012). Metabolism of reactive oxygen species and reactive nitrogen species in pepper (Capsicum annuum l.) plants under low temperature stress. Plant Cell Environ. 35, 281–295. doi: 10.1111/j.1365-3040.2011.02310.x 21414013

[B6] Akaike T. Okamoto S. Sawa T. Yoshitake J. Tamura F. Ichimori K.. (2003). 8-nitroguanosine formation in viral pneumonia and its implication for pathogenesis. Proc. Natl. Acad. Sci. U. S. A. 100, 685–690. doi: 10.1073/pnas.0235623100 12522148 PMC141057

[B7] Albertos P. Romero-Puertas M. C. Tatematsu K. Mateos I. Sánchez-Vicente I. Nambara E.. (2015). S-nitrosylation triggers ABI5 degradation to promote seed germination and seedling growth. Nat. Commun. 6, 1–10. doi: 10.1038/ncomms9669 PMC4639896 26493030

[B8] Alché J. D. D. (2019). A concise appraisal of lipid oxidation and lipoxidation in higher plants. Redox Biol. 23, 101136. doi: 10.1016/j.redox.2019.101136 30772285 PMC6859586

[B9] Alp K. Terzi H. Yildiz M. (2022). Proteomic and physiological analyses to elucidate nitric oxide-mediated adaptive responses of barley under cadmium stress. Physiol. Mol. Biol. Plants 28, 1467–1476. doi: 10.1007/s12298-022-01214-3 36051236 PMC9424405

[B10] Apel K. Hirt H. (2004). Reactive oxygen species: metabolism, oxidative stress and signal transduction. Annu. Rev. Plant Biol. 55, 373–399. doi: 10.1146/annurev.arplant.55.031903.141701 15377225

[B11] Aranda-Caño L. Sánchez-Calvo B. Begara-Morales J. C. Chaki M. Mata-Pérez C. Padilla M. N.. (2019). Post-translational modification of proteins mediated by nitro-fatty acids in plants: nitroalkylation. Plants (basel) 8 (4), 82. doi: 10.3390/plants8040082 30934982 PMC6524050

[B12] Aranda-Caño L. Valderrama R. Pedrajas J. R. Begara-Morales J. C. Chaki M. Padilla M. N.. (2022). Nitro-oleic acid-mediated nitroalkylation modulates the antioxidant function of cytosolic peroxiredoxin Tsa1 during heat stress in saccharomyces cerevisiae. Antioxidants 11, 972. doi: 10.3390/antiox11050972 35624836 PMC9137801

[B13] Arimura G. I. Kost C. Boland W. (2005). Herbivore-induced, indirect plant defences. Biochim. Et Biophys. Acta (BBA) - Mol. Cell Biol. Lipids 1734 (2), 91–111. doi: 10.1016/j.bbalip.2005.03.001 15904867

[B14] Arruebarrena Di Palma A. Di Fino L. M. Salvatore S. R. D’Ambrosio J. M. Garćıa-Mata C. Schopfer F. J.. (2020). Nitro-oleic acid triggers ROS production *via* NADPH oxidase activation in plants: A pharmacological approach. J. Plant Physiol. 246-247, 153128. doi: 10.1016/j.jplph.2020.153128 32065921 PMC7153499

[B15] Asgher M. Per T. S. Masood A. Fatma M. Freschi L. Corpas F. J.. (2017). Nitric oxide signaling and its crosstalk with other plant growth regulators in plant responses to abiotic stress. Environ. Sci. pollut. Res. 24, 2273–2285. doi: 10.1007/s11356-016-7947-8 27812964

[B16] Astier J. Kulik A. Koen E. Besson-Bard A. Bourque S. Jeandroz S.. (2012). Protein s-nitrosylation: What's going on in plants? Free Radic. Biol. Med. 53, 1101–1110. doi: 10.1016/j.freeradbiomed.2012.06.032 22750205

[B17] Astier J. Rasul S. Koen E. Manzoor H. Besson-Bard A. Lamotte O.. (2011). S-nitrosylation: An emerging post-translational protein modification in plants. Plant Sci. 181, 527–533. doi: 10.1016/j.plantsci.2011.02.011 21893248

[B18] Babbar R. Karpinska B. Grover A. Foyer C. H. (2021). Heat-induced oxidation of the nuclei and cytosol. Front. Plant Sci. 11. doi: 10.3389/fpls.2020.617779 PMC7835529 33510759

[B19] Bailey-Serres J. Voesenek L. A. C. J. (2008). Flooding stress: Acclimations and genetic diversity. Annu. Rev. Plant Biol. 59, 313–339. doi: 10.1146/annurev.arplant.59.032607.092752 18444902

[B20] Baker L. M. Baker P. R. Golin-Bisello F. Schopfer F. J. Fink M. Woodcock S. R.. (2007). Nitro-fatty acid reaction with glutathione and cysteine. kinetic analysis of thiol alkylation by a Michael addition reaction. J. Biol. Chem. 282, 31085–31093. doi: 10.1074/jbc.M704085200 17720974 PMC2169496

[B21] Begara-Morales J. C. Chaki M. Valderrama R. Sanchez-Calvo B. Mata-Perez C. Padilla M. N.. (2018). Nitric oxide buffering and conditional nitric oxide release in stress response. J. Exp. Bot. 69, 3425–3438. doi: 10.1093/jxb/ery072 29506191

[B22] Begara-Morales J. C. Sánchez-Calvo B. Chaki M. Valderrama R. Mata-Pérez C. López-Jaramillo J.. (2014). Dual regulation of cytosolic ascorbate peroxidase (APX) by tyrosine nitration and s-nitrosylation. J. Exp. Bot. 65:2, 527–538. doi: 10.1093/jxb/ert396 24288182 PMC3904709

[B23] Bolwell G. P. Wojtaszek P. (1997). Mechanisms for the generation of reactive oxygen species in plant defence–a broad perspective. Physiol. Mol. Plant Pathol. 51, 347–366. doi: 10.1006/pmpp.1997.0129

[B24] Brosché M. Merilo E. Mayer F. Pechter P. Puz~orjova I. Brader G.. (2010). Natural variation in ozone sensitivity among arabidopsis thaliana accessions and its relation to stomatal conductance. Plant Cell Environ. 33, 914–925. doi: 10.1111/j.1365-3040.2010.02116.x 20082669

[B25] Brown G. C. (2001). Regulation of mitochondrial respiration by nitric oxide inhibition of cytochrome c oxidase. Biochim. Biophys. Acta BBA - Bioenerg. 1504, 46–57. doi: 10.1016/S0005-2728(00)00238-3 11239484

[B26] Brown B. A. Jenkins G. I. (2008). UV-B signaling pathways with different fluence-rate response profiles are distinguished in mature arabidopsis leaf tissue by requirement for UVR8, HY5 and HYH. Plant Physiol. 146:2, 576–588. doi: 10.1104/pp.107.108456 18055587 PMC2245850

[B27] Cai W. Liu W. Wang W. S. Fu Z. W. Han T. T. Lu Y. T. (2015). Overexpression of rat neurons nitric oxide synthase in rice enhances drought and salt tolerance. PloS One 10 (6), e0131599. doi: 10.1371/journal.pone.0131599 26121399 PMC4485468

[B28] Caldwell M. M. Ballaré C. L. Bornman J. F. Flint S. D. Björn L. O. Teramura A. H.. (2003). Terrestrial ecosystems, increased solar ultraviolet radiation and interactions with other climatic change factors. Photochem. Photobiol. Sci. 2:1, 29–38. doi: 10.1039/b211159b 12659537

[B29] Camejo D. del Carmen Romero-Puertas M. Rodríguez-Serrano M. Sandalio L. M. Lázaro J. J. Jiménez A.. (2013). Salinity-induced changes in s-nitrosylation of pea mitochondrial proteins. J. Proteomics 79, 87–99. doi: 10.1016/j.jprot.2012.12.003 23238061

[B30] Camejo D. Ortiz-Espín A. Lázaro J. J. Romero-Puertas M. C. Lázaro-Payo A. Sevilla F.. (2015). Functional and structural changes in plant mitochondrial PrxII f caused by NO. J. Proteomics 119, 112–125. doi: 10.1016/j.jprot.2015.01.022 25682994

[B31] Cassia R. Amenta M. Fernández M. B. Nocioni M. Dávila V. (2019). “The role of nitric oxide in the antioxidant defense of plants exposed to UV-b radiation,” in Reactive oxygen, nitrogen and sulfur species in plants: production, metabolism, signaling and defense mechanisms, 555–572. doi: 10.1002/9781119468677.ch22

[B32] Castillo M. Lozano-Juste J. González-Guzmán M. Rodriguez L. Rodriguez P. L. León J. (2015). Inactivation of PYR/PYL/RCAR ABA receptors by tyrosine nitration may enable rapid inhibition of ABA signaling by nitric oxide in plants. Sci. Signaling 8, ra89. doi: 10.1126/scisignal.aaa7981 26329583

[B33] Chaki M. Carreras A. López-Jaramillo J. Begara-Morales J. C. Sánchez-Calvo B. Valderrama R.. (2013). Tyrosine nitration provokes inhibition of sunflower carbonic anhydrase (β-CA) activity under high temperature stress. Nitric. Oxide 29, 30–33. doi: 10.1016/j.niox.2012.12.003 23266784

[B34] Chaki M. Valderrama R. Fernández-Ocaña A. M. Carreras A. Gómez-Rodríguez M. V. López-Jaramillo J.. (2011). High temperature triggers the metabolism of s-nitrosothiols in sunflower mediating a process of nitrosative stress which provokes the inhibition of ferredoxin-NADP reductase by tyrosine nitration. Plant Cell Environ. 34, 1803–1818. doi: 10.1111/j.1365-3040.2011.02376.x 21676000

[B35] Chaki M. Valderrama R. Fernández-Ocaña A. M. Carreras A. Gómez-Rodríguez M. V. Pedrajas J. R.. (2010). Mechanical wounding induces a nitrosative stress by down-regulation of GSNO reductase and an increase in s-nitrosothiols in sunflower (Helianthus annuus) seedlings. J. Exp. Bot. 62:6, 1803–1813. doi: 10.1093/jxb/erq358 21172815 PMC3060671

[B36] Chen K. Chen L. Fan J. Fu J. (2013). Alleviation of heat damage to photosystem II by nitric oxide in tall fescue. Photosynth. Res. 116, 21–31. doi: 10.1007/s11120-013-9883-5 23832593

[B37] Chen R. Sun S. Wang C. Li Y. Liang Y. An F.. (2009). The arabidopsis PARAQUAT RESISTANT2 gene encodes an s-nitrosoglutathione reductase that is a key regulator of cell death. Cell Res. 19, 1377–1387. doi: 10.1038/cr.2009.117 19806166

[B38] Clark D. Durner J. Navarre D. A. Klessig D. F. (2000). Nitric oxide inhibition of tobacco catalase and ascorbate peroxidase. Mol. Plant Microbe Interact. 13 (12), 1380–1384. doi: 10.1094/MPMI.2000.13.12.1380 11106031

[B39] Contreras R. A. Pizarro M. Köhler H. Sáez C. A. Zúñiga G. E. (2018). Copper stress induces antioxidant responses and accumulation of sugars and phytochelatins in Antarctic colobanthus quitensis (Kunth) bartl. Biol. Res. 51, 48. doi: 10.1186/S40659-018-0197-0 30428921 PMC6234666

[B40] Corpas F. J. Barroso J. B. (2014). Peroxynitrite (ONOO–) is endogenously produced in arabidopsis peroxisomes and is overproduced under cadmium stress. Ann. Bot. 113, 87–96. doi: 10.1093/AOB/MCT260 24232384 PMC3864731

[B41] Corpas F. J. Chaki M. Fernández-Ocaña A. Valderrama R. Palma J. M. Carreras A.. (2008). Metabolism of reactive nitrogen species in pea plants under abiotic stress conditions. Plant Cell Physiol. 49, 1711–1722. doi: 10.1093/pcp/pcn144 18801763

[B42] David A. Yadav S. Baluška F. Bhatla S. C. (2015). Nitric oxide accumulation and protein tyrosine nitration as a rapid and long distance signalling response to salt stress in sunflower seedlings. Nitric. Oxide 50, 28–37. doi: 10.1016/j.niox.2015.08.003 26296694

[B43] del Río L. A. (2015). ). ROS and RNS in plant physiology: an overview. J. Exp. Bot. 66, 2827–2837. doi: 10.1093/jxb/erv099 25873662

[B44] Demecsová L. Tamás L. (2019). Reactive oxygen species, auxin and nitric oxide in metal-stressed roots: toxicity or defence. BioMetals 32(5): 717–744. doi: 10.1007/S10534-019-00214-3 31541378

[B45] Dietz K. J. (2008). Redox signal integration: from stimulus to networks and genes. Physiol. Plant 133, 459–468. doi: 10.1111/j.1399-3054.2008.01120.x 18429942

[B46] Di Fino L. M. Cerrudo I. Salvatore S. R. Schopfer F. J. García-Mata C. Laxalt A. M. (2020). Exogenous nitro-oleic acid treatment inhibits primary root growth by reducing the mitosis in the meristem in arabidopsis thaliana. Front. Plant Sci. 11. doi: 10.3389/fpls.2020.01059 PMC7385231 32793255

[B47] Domingos P. Prado A. M. Wong A. Gehring C. Feijo J. A. (2015). Nitric oxide: A multitasked signaling gas in plants. Mol. Plant 8, 506–520. doi: 10.1016/j.molp.2014.12.010 25680232

[B48] Drew M. C. He C.-J. Morgan P. W. (2000). Programmed cell death and aerenchyma formation in roots. Trends Plant Sci. 5, 123–127. doi: 10.1016/S1360-1385(00)01570-3 10707078

[B49] Duan S. W. Wang J. J. Gao C. H. Jin C. Y. Li D. Peng D. S.. (2018). Functional characterization of a heterologously expressed brassica napus WRKY41-1 transcription factor in regulating anthocyanin biosynthesis in arabidopsis thaliana. Plant Sci. 268, 47–53. doi: 10.1016/j.plantsci.2017.12.010 29362083

[B50] Duruibe J. Ogwuegbu M. Egwurugwu J. (2007). Heavy metal pollution and human biotoxic effects. Int. J. Phys. Sci. 2, 112–118. doi: 10.5897/IJPS.9000289

[B51] Ederli L. Morettini R. Borgogni A. Wasternack C. Miersch O. Reale L.. (2006). Interaction between nitric oxide and ethylene in the induction of alternative oxidase in ozone-treated tobacco plants. Plant Physiol. 142, 595–608. doi: 10.1104/pp.106.085472 16935990 PMC1586042

[B52] Egbichi M. Keyster N. Ludidi. (2014). Effect of exogenous application of nitric oxide on salt stress responses of soybean. South Afr. J. Bot. 90, 131–136. doi: 10.1016/j.sajb.2013.11.002

[B53] Faine L. A. Cavalcanti D. M. Rudnicki M. Ferderbar S. Macedo S. M. Souza H. P.. (2010). Bioactivity of nitrolinoleate: effects on adhesion molecules and CD40eCD40L system. J. Nutr. Biochem. 21 (2), 125–132. doi: 10.1016/j.jnutbio.2008.12.004 19195864

[B54] Fancy N. N. Bahlmann A. K. Loake G. J. (2017). Nitric oxide function in plant abiotic stress. Plant Cell Environ. 40, 462–472. doi: 10.1111/pce.12707 26754426

[B55] Farmer E. E. Ryan C. A. (1992). Octadecanoid precursors of jasmonic acid activate the synthesis of wound-inducible proteinase inhibitors. Plant Cell 4 (2), 129–134. doi: 10.1105/tpc.4.2.129 12297644 PMC160114

[B56] Fatma M. Masood A. Per T. S. Khan N. A. (2016). Nitric oxide alleviates salt stress inhibited photosynthetic performance by interacting with sulfur assimilation in mustard. Front. Plant Sci. 7. doi: 10.3389/fpls.2016.00521 PMC4842777 27200007

[B57] Favory J. J. Stec A. Gruber H. Rizzini L. Oravecz A. Funk M.. (2009). Interaction of COP1 and UVR8 regulates UV-b induced photomorfogenesys and stress acclimatation in arabidopsis. EMBO J. 28:5, 591–601. doi: 10.1038/emboj.2009.4 19165148 PMC2657586

[B58] Feigl G. Kolbert Z. Lehotai N. Molnár Á. Ördög A. Bordé Á.. (2016). Different zinc sensitivity of brassica organs is accompanied by distinct responses in protein nitration level and pattern. Ecotoxicol. Environ. Saf. 125, 141–152. doi: 10.1016/J.ECOENV.2015.12.006 26685787

[B59] Finkelstein R. R. Gampala S. S. Rock C. D. (2002). Abscisic acid signaling in seeds and seedlings. Plant Cell. 14 Suppl (Suppl), S15–S45. doi: 10.1105/tpc.010441 12045268 PMC151246

[B60] Fiscus E. L. Booker F. L. Burkey K. O. (2005). Crop responses to ozone: uptake, modes of action, carbon assimilation and partitioning. Plant Cell Environ. 28, 997–1011. doi: 10.1111/j.1365-3040.2005.01349.x

[B61] Freschi L. (2013). Nitric oxide and phytohormone interactions: Current status and perspectives. Front. Plant Sci. 4. doi: 10.3389/fpls.2013.00398 PMC3793198 24130567

[B62] Gao X. Ma J. Tie J. Li Y. Hu L. Yu J. (2022). BR-Mediated protein s-nitrosylation alleviated low-temperature stress in mini Chinese cabbage (Brassica rapa ssp. pekinensis) Int. J. Mol. Sci. 23, 10964. doi: 10.3390/ijms231810964 36142872 PMC9503245

[B63] Gaupels F. Furch A. C. U. Zimmermann M. R. Chen F. Kaever V. Buhtz A.. (2016). Systemic induction of NO-, redox-, and cGMP signaling in the pumpkin extrafascicular phloem upon local leaf wounding. Front. Plant Sci. 7. doi: 10.3389/fpls.2016.00154 PMC4751408 26904092

[B64] Gaupels F. Kuruthukulangarakoola G. T. Durner J. (2011). Upstream and downstream signals of nitric oxide in pathogen defence. Curr. Opin. Plant Biol. 14, 707–714. doi: 10.1016/j.pbi.2011.07.005 21816662

[B65] Geisler A. C. Rudolph T. K. (2012). Nitroalkylation a redox sensitive signaling pathway. Biochem. Biophys. Acta 1820 (6), 777–784. doi: 10.1016/j.bbagen.2011.06.014 21723375

[B66] Gill S. S. Hasanuzzaman M. Nahar K. Macovei A. Tuteja N. (2013). Importance of nitric oxide in cadmium stress tolerance in crop plants. Plant Physiol. Biochem. 63, 254–261. doi: 10.1016/J.PLAPHY.2012.12.001 23313792

[B67] González-Gordo S. Rodríguez-Ruiz M. López-Jaramillo J. Muñoz-Vargas M. A. Palma J. M. Corpas F. J. (2022). Nitric oxide (NO) differentially modulates the ascorbate peroxidase (APX) isozymes of sweet pepper (Capsicum annuum l.) fruits. Antioxidants 11, 765. doi: 10.3390/antiox11040765 35453450 PMC9029456

[B68] Gorczynski M. J. Huang J. Lee H. King S. B. (2007). Evaluation of nitroalkenes as nitric oxide donors. Bioorg. med. Chem. Lett. 17, 2013–2017. doi: 10.1016/j.bmcl.2007.01.016 17270440

[B69] Gupta K. J. Igamberdiev A. U. (2016). Reactive nitrogen species in mitochondria and their implications in plant energy status and hypoxic stress tolerance. Front. Plant Sci. 7. doi: 10.3389/fpls.2016.00369 PMC4806263 27047533

[B70] Gupta K. J. Shah J. K. Brotman Y. Jahnke K. Willmitzer L. Kaiser W. M.. (2012). Inhibition of aconitase by nitric oxide leads to induction of the alternative oxidase and to a shift of metabolism towards biosynthesis of amino acids. J. Exp. Bot. 63, 1773–1784. doi: 10.1093/jxb/ers053 22371326

[B71] Gupta K. J. Zabalza A. Van Dongen J. T. (2009). Regulation of respiration when the oxygen availability changes. Physiol. Plant 137, 383–391. doi: 10.1111/j.1399-3054.2009.01253.x 19549068

[B72] Halliwell B. (2006). Reactive species and antioxidants. redox biology is a fundamental theme of aerobic life. Plant Physiol. 141, 312–322. doi: 10.1104/pp.106.077073 16760481 PMC1475431

[B73] Hamurcu M. Khan M. K. Pandey A. Ozdemir C. Avsaroglu Z. Z. Elbasan F.. (2020). Nitric oxide regulates watermelon (Citrullus lanatus) responses to drought stress. 3 Biotech. 10 (11), 494. doi: 10.1007/s13205-020-02479-9 PMC7593377 33134012

[B74] Hasan M. Rahman M. Skalicky M. Alabdallah N. M. Waseem M. Jahan M. S.. (2021). Ozone induced stomatal regulations, MAPK and phytohormone signaling in plants. Intl J. Mol. Sci. 22 (12), 6304. doi: 10.3390/ijms22126304 PMC8231235 34208343

[B75] Hawkes S. J. (1997). What is a “Heavy metal”? J. Chem. Educ. 74, 1374. doi: 10.1021/ed074p1374

[B76] He N. Y. Chen L. S. Sun A. Z. Zhao Y. Yin S. N. Guo F. Q. (2022). A nitric oxide burst at the shoot apex triggers a heat-responsive pathway in arabidopsis. Nat. Plants 8, 434–450. doi: 10.1038/s41477-022-01135-9 35437002

[B77] He H. Y. He L. F. Gu M. H. Li X. F. (2012). Nitric oxide improves aluminum tolerance by regulating hormonal equilibrium in the root apices of rye and wheat. Plant Sci. 183, 123–130. doi: 10.1016/J.PLANTSCI.2011.07.012 22195585

[B78] He J. M. Ma X. G. Zhang Y. Sun T. F. Xu F. F. Chen Y. P.. (2013). Role and interrelationship of gα protein, hydrogen peroxide, and nitric oxide in ultraviolet b-induced stomatal closure in arabidopsis leaves. Plant Physiol. 161 (3), 1570–1583. doi: 10.1104/pp.112.211623 23341360 PMC3585617

[B79] Hebelstrup K. H. Møller I. M. (2015). “Mitochondrial signaling in plants under hypoxia: Use of reactive oxygen species (ROS) and reactive nitrogen species (RNS),” in Reactive oxygen and nitrogen species signaling and communication in plants signaling and communication in plants. Eds. Gupta K. J. Igamberdiev A. U. (Cham: Springer International Publishing), 63–77. doi: 10.1007/978-3-319-10079-1_4

[B80] Hebelstrup K. H. van Zanten M. Mandon J. Voesenek L. A. C. J. Harren F. J. M. Cristescu S. M.. (2012). Haemoglobin modulates NO emission and hyponasty under hypoxia-related stress in arabidopsis thaliana. J. Exp. Bot. 63, 5581–5591. doi: 10.1093/jxb/ers210 22915746 PMC3444272

[B81] Hess D. T. Matsumoto A. Kim S. O. Marshall H. E. Stamler J. S. (2005). Protein s-nitrosylation: Purview and parameters. Nat. Rev. Mol. Cell Biol. 6, 150–166. doi: 10.1038/nrm1569 15688001

[B82] Hoang X. L. T. Nhi D. N. H. Thu N. B. A. Thao N. P. Tran L. P. (2017). Transcription factors and their roles in signal transduction in plants under abiotic stresses. Curr. Genomics 18, 483–497. doi: 10.2174/1389202918666170227150057 29204078 PMC5684650

[B83] Honda K. Yamada N. Yoshida R. Ihara H. Sawa T. Akaike T.. (2015). 8-mercapto-cyclic GMP mediates hydrogen sulfide-induced stomatal closure in arabidopsis. Plant Cell Physiol. 56, 1481–1489. doi: 10.1093/pcp/pcv069 25975264

[B84] Hoque M. N. Tahjib-Ul-arif M. Hannan A. Sultana N. Akhter S. Hasanuzzaman M.. (2021). Melatonin modulates plant tolerance to heavy metal stress: Morphological responses to molecular mechanisms. Int. J. Mol. Sci. 22 (21), 11445. doi: 10.3390/IJMS222111445 34768875 PMC8584185

[B85] Horak H. Sierla M. Wang C. Wang Y. S. Nuhkat M. Valk E.. (2016). A dominant mutation in the ht1 kinase uncovers roles of MAP kinases and GHR1 in CO2-induced stomatal closure. Plant Cell 28, 2493–2509. doi: 10.1105/tpc.16.00131 27694184 PMC5134974

[B86] Hossain M. A. Piyatida P. Teixeira Da Silva J. A. Fujita M. (2012). Molecular mechanism of heavy metal toxicity and tolerance in plants: central role of glutathione in detoxification of reactive oxygen species and methylglyoxal. J. od Bot. 2012, 1–37. doi: 10.1155/2012/872875

[B87] Houmani H. Rodríguez-Ruiz M. Palma J. M. Corpas F. J. (2018). Mechanical wounding promotes local and long distance response in the halophyte cakile maritima through the involvement of the ROS and RNS metabolism. Nitric. Oxide 74, 93–101. doi: 10.1016/j.niox.2017.06.008 28655650

[B88] Igamberdiev A. U. Baron K. Manac’h-Little N. Stoimenova M. Hill R. D. (2005). The Haemoglobin/Nitric oxide cycle: Involvement in flooding stress and effects on hormone signalling. Ann. Bot. 96, 557–564. doi: 10.1093/aob/mci210 16027133 PMC4247025

[B89] Ihara H. Sawa T. Nakabeppu Y. Akaike T. (2011). Nucleotides function as endogenous chemical sensors for oxidative stress signaling. J. Clin. Biochem. Nutr. 48, 33–39. doi: 10.3164/jcbn.11-003FR 21297909 PMC3022061

[B90] Jain P. Bhatla S. C. (2018). Tyrosine nitration of cytosolic peroxidase is probably triggered as a long distance signaling response in sunflower seedling cotyledons subjected to salt stress. PloS One 13, e0197132. doi: 10.1371/journal.pone.0197132 29768452 PMC5955538

[B91] Jain P. von Toerne C. Lindermayr C. Bhatla S. C. (2018). S-nitrosylation/denitrosylation as a regulatory mechanism of salt stress sensing in sunflower seedlings. Physiol. Plant. 162, 49–72. doi: 10.1111/ppl.12641 28902403

[B92] Jayawardhane J. Cochrane D. W. Vyas P. Bykova N. V. Vanlerberghe G. C. Igamberdiev A. U. (2020). Roles for plant mitochondrial alternative oxidase under normoxia, hypoxia, and reoxygenation conditions. Front. Plant Sci. 11. doi: 10.3389/fpls.2020.00566 PMC7243820 32499803

[B93] Jethva J. Schmidt R. R. Sauter M. Selinski J. (2022). Try or die: Dynamics of plant respiration and how to survive low oxygen conditions. Plants 11, 205. doi: 10.3390/plants11020205 35050092 PMC8780655

[B94] Joudoi T. Shichiri Y. Kamizono N. Akaike T. Sawa T. Yoshitake J.. (2013). Nitrated cyclic GMP modulates guard cell signaling in arabidopsis. Plant Cell 25, 558–571. doi: 10.1105/tpc.112.105049 23396828 PMC3608778

[B95] Kabange N. R. Mun B. G. Lee S. M. Kwon Y. Lee D. Lee G. M.. (2022). Nitric oxide: A core signaling molecule under elevated GHGs (CO2, CH4, N2O, O3)-mediated abiotic stress in plants. Front. Plant Sci. 13. doi: 10.3389/fpls.2022.994149 PMC9667792 36407609

[B96] Kaiserli E Jenkins GI (2007). UV-B promotes rapid nuclear translocation of the UV-B-specific signaling component UVR8 and activates its function in the nucleus. Plant Cell 19 (8), 2662–73. 17720867 10.1105/tpc.107.053330 PMC2002606

[B97] Kandziora-Ciupa M. Ciepał R. Nadgórska-Socha A. Barczyk G. (2013). A comparative study of heavy metal accumulation and antioxidant responses in vaccinium myrtillus l. leaves in polluted and non-polluted areas. Environ. Sci. pollut. Res. 20, 4920–4932. doi: 10.1007/S11356-012-1461-4 PMC3695683 23319337

[B98] Kaya C. Ashraf M. Alyemeni M. N. Ahmad P. (2020). Responses of nitric oxide and hydrogen sulfide in regulating oxidative defence system in wheat plants grown under cadmium stress. Physiol. Plant 168, 345–360. doi: 10.1111/PPL.13012 31343742

[B99] Kende H. van der Knaap E. Cho H.-T. (1998). Deepwater rice: A model plant to study stem Elongation1. Plant Physiol. 118, 1105–1110. doi: 10.1104/pp.118.4.1105 9847084 PMC1539197

[B100] Keyster M. Klein A. Egbichi I. Jacobs A. Ludidi N. (2011). Nitric oxide increases the enzymatic activity of three ascorbate peroxidase isoforms in soybean root nodules. Plant Signaling Behav. 6, 956–961. doi: 10.4161/psb.6.7.14879 PMC3257769 21494099

[B101] Khan M. Y. Prakash V. Yadav V. Chauhan D. K. Prasad S. M. Ramawat N.. (2019). Regulation of cadmium toxicity in roots of tomato by indole acetic acid with special emphasis on reactive oxygen species production and their scavenging. Plant Physiol. Biochem. 142, 193–201. doi: 10.1016/J.PLAPHY.2019.05.006 31301530

[B102] Kliebenstein D. J. Lim J. E. Landry L. G. Last R. L. (2002). Arabidopsis UVR8 regulates ultraviolet-b signal transduction and tolerance and contains sequence similarity to human regulator of chromatin condensation 1. Plant Physiol. 30 (1), 234–243. doi: 10.1104/pp.005041 PMC166556 12226503

[B103] Kolbert Z. Molnár Á. Szollosi R. Feigl G. Erdei L. Ördög A. (2018). Nitro-oxidative stress correlates with Se tolerance of astragalus species. Plant Cell Physiol. 59, 1827–1843. doi: 10.1093/PCP/PCY099 29800274

[B104] Kolbert Z. Molnï Ï. Olï D. Feigl G. HorvÏ E. Erdei L.. (2019). S-nitrosothiol signaling is involved in regulating hydrogen peroxide metabolism of zinc-stressed arabidopsis. Plant Cell Physiol. 60 (11), 2449–2463. doi: 10.1093/pcp/pcz138 31340034

[B105] Kolbert Z. Petõ A. Lehotai N. Feigl G. Erdei L. (2012). Long-term copper (Cu 2+ ) exposure impacts on auxin, nitric oxide (NO) metabolism and morphology of arabidopsis thaliana l. Plant Growth Regul. 68, 151–159. doi: 10.1007/s10725-012-9701-7

[B106] Kontunen-Soppela S. Parviainen J. Ruhanen H. Brosché M. Keinänen M. Thakur R. C.. (2010). Gene expression responses of paper birch (Betula papyrifera) to elevated CO2 and O3 during leaf maturation and senescence. Environ. pollut. 158, 959–968. doi: 10.1016/j.envpol.2009.10.008 19889492

[B107] Krasylenko Y. A. Yemets A. I. Sheremet Y. A. Blume Y. B. (2012). Nitric oxide as a critical factor for perception of UV-b irradiation by microtubules in arabidopsis. Physiol. Plant 145 (4), 505–515. doi: 10.1111/j.1399-3054.2011.01530.x 21973209

[B108] Lamotte O. Bertoldo J. B. Besson-Bard A. Rosnoblet C. Aime S. Hichami S.. (2014). Protein s-nitrosylation: Specificity and identification strategies in plants. Front. Chem. 2. doi: 10.3389/fchem.2014.00114 PMC4285867 25750911

[B109] Lamotte O. Bertoldo J. B. Besson-Bard A. Rosnoblet C. Aimé S. Hichami S.. (2015). Protein s-nitrosylation: specificity and identification strategies in plants. Front. Chem. 2. doi: 10.3389/fchem.2014.00114 PMC4285867 25750911

[B110] Lee U. Wie C. Fernandez B. O. Feelisch M. Vierling E. (2008). Modulation of nitrosative stress by s-nitrosoglutathione reductase is critical for thermotolerance and plant growth in arabidopsis. Plant Cell 20, 786–802. doi: 10.1105/tpc.107.052647 18326829 PMC2329944

[B111] Leterrier M. Airaki M. Palma J. M. Chaki M. Barroso J. B. Corpas F. J. (2012). Arsenic triggers the nitric oxide (NO) and s-nitrosoglutathione (GSNO) metabolism in arabidopsis. Environ. pollut. 166, 136–143. doi: 10.1016/J.ENVPOL.2012.03.012 22504427

[B112] Li X. Liu Z. Ren H. Kundu M. Zhong F. W. Wang L.. (2022). Dynamics and mechanism of dimer dissociation of photoreceptor UVR8. Nat. Commun. 13 (1), 93. doi: 10.1038/s41467-021-27756-w 35013256 PMC8748919

[B113] Li X. Pan Y. Chang B. Wang Y. Tang Z. (2016). NO promotes seed germination and seedling growth under high salt may depend on EIN3 protein in arabidopsis. Front. Plant Sci. 6. doi: 10.3389/fpls.2015.01203 PMC4703817 26779234

[B114] Li Z. Wakao S. Fischer B. B. Niyogi K. K. (2009). Sensing and responding to excess light. Annu. Rev. Plant Biol. 60, 239–260. doi: 10.1146/annurev.arplant.58.032806.103844 19575582

[B115] Lima E. S. Bonini M. G. Augusto O. Barbeiro H. V. Souza H. P. Abdalla D. S. (2005). Nitrated lipids decompose to nitric oxide and lipid radicals and cause vasorelaxation. Free Radic. Biol. Med. 39 (4), 532–539. doi: 10.1016/j.freeradbiomed.2005.04.005 16043024

[B116] Lin C. C. Jih P. J. Lin H. H. Lin J. S. Chang L. L. Shen Y. H.. (2011). Nitric oxide activates superoxide dismutase and ascorbate peroxidase to repress the cell death induced by wounding. Plant Mol. Biol. 77, 235–249. doi: 10.1007/s11103-011-9805-x 21833542

[B117] Liu T. Xu J. Li J. Hu X. (2019). NO is involved in JA- and H2O2-mediated ALA-induced oxidative stress tolerance at low temperatures in tomato. Environ. Exp. Bot. 161, 334–343. doi: 10.1016/j.envexpbot.2018.10.020

[B118] Lopez-Molina L. Chua N. H. (2000). A null mutation in a bZIP factor confers ABA-insensitivity in arabidopsis thaliana. Plant Cell Physiol. 41 (5), 541–547. doi: 10.1093/pcp/41.5.541 10929936

[B119] Ma N. Adachi Y. Hiraku Y. Horiki N. Horiike S. Imoto I.. (2004). Accumulation of 8-nitroguanine in human gastric epithelium induced by helicobacter pylori infection. Biochem. Biophys. Res. Commun. 319, 506–510. doi: 10.1016/j.bbrc.2004.04.193 15178435

[B120] Mackerness S. A. H. John C. F. Jordan B. Thomas B. (2001). Early signaling componenets in ultraviolet-b responses: distinct roles for different reactive oxygen species and nitric oxide. FEBS Lett. 489 (2-3), 237–242. doi: 10.1016/s0014-5793(01)02103-2 11165257

[B121] Manasa S. L. Panigrahy M. Panigrahi K. C. S. Rout G. R. (2022). Overview of cold stress regulation in plants. Bot. Rev. 88, 359–387. doi: 10.1007/s12229-021-09267-x

[B122] Manrique-Gil I. Sánchez-Vicente I. Torres-Quezada I. Lorenzo O. (2021). Nitric oxide function during oxygen deprivation in physiological and stress processes. J. Exp. Bot. 72, 904–916. doi: 10.1093/jxb/eraa442 32976588 PMC7876777

[B123] Mata-Pérez C. Begara-Morales J. C. Chaki M. Sánchez-Calvo B. Valderrama R. Padilla M. N.. (2016c). Protein tyrosine nitration during development and abiotic stress response in plants. Front. Plant Sci. 7. doi: 10.3389/fpls.2016.01699 PMC5108813 27895655

[B124] Mata-Pérez C. Sánchez-Calvo B. Begara-Morales J. C. Carreras A. Padilla M. N. Melguizo M.. (2016b). Nitro-linolenic acid is a nitric oxide donor. Nitric. Oxide 57, 57–63. doi: 10.1016/j.niox.2016b.05.003 27164295

[B125] Mata-Pérez C. Sánchez-Calvo B. Padilla M. N. Begara-Morales J. C. Luque F. Melguizo M.. (2016a). Nitro-fatty acids in plant signaling: Nitro-linolenic acid induces the molecular chaperone network in arabidopsis. Plant Physiol. 170, 686–701. doi: 10.1104/pp.15.01671 26628746 PMC4734579

[B126] Mengel A. Chaki M. Shekariesfahlan A. Lindermayr C. (2013). Effect of nitric oxide on gene transcription – s-nitrosylation of nuclear proteins. Front. Plant Sci. 4. doi: 10.3389/fpls.2013.00293 PMC3729996 23914201

[B127] Millar A. H. Day D. A. (1996). Nitric oxide inhibits the cytochrome oxidase but not the alternative oxidase of plant mitochondria. FEBS Lett. 398, 155–158. doi: 10.1016/S0014-5793(96)01230-6 8977097

[B128] Mills G. Sharps K. Simpson D. Pleijel H. Broberg M. Uddling J.. (2018). Ozone pollution will compromise efforts to increase global wheat production. Global Change Biol. 24, 3560–3574. doi: 10.1111/gcb.14157 29604158

[B129] Mittler R. Vanderauwera S. Suzuki N. Miller G. Tognetti V. B. Vandepoele K.. (2011). ROS signaling: the new wave? Trends Plant Sci. 16 (6), 300–309. doi: 10.1016/j.tplants.2011.03.007 21482172

[B130] Morales L. O. Shapiguzov A. Safronov O. Lepälä J. Vaahtera L. Yarmolinsky D.. (2021). Ozone responses in arabidopsis: beyond stomatal conductance. Plant Physiol. 186, 180–192. doi: 10.1093/plphys/kiab097 33624812 PMC8154098

[B131] Moreau M. Lindermayr C. Durner J. Klessig D. F. (2010). NO synthesis and signaling in plants–where do we stand? Physiol. Plant. 138, 372–383. doi: 10.1111/j.1399-3054.2009.01308.x 19912564

[B132] Mostafa S. Wang Y. Zeng W. Jin B. (2022). Plant responses to herbivory, wounding, and infection. Int. J. Mol. Sci. 23 (13), 7031. doi: 10.3390/ijms23137031 35806046 PMC9266417

[B133] Mukherjee S. (2022). “Atmospheric nitric oxide (NO) regulates ozone (O3)-induced stress signaling in plants: Ally or foe?,” in Plant stress: Challenges and management in the new decade (Cham, Switzerland: Springer), 89–96. doi: 10.1007/978-3-030-95365-2_5

[B134] Nagajyoti P. C. Lee K. D. Sreekanth T. V. M. (2010). Heavy metals, occurrence and toxicity for plants: a review. Environ. Chem. Letters. 8 (3), 199–216. doi: 10.1007/S10311-010-0297-8

[B135] Nagatoshi Y. Mitsuda N. Hayashi M. Inoue S. Okuma E. Kubo A.. (2016). GOLDEN 2-LIKE transcription factors for chloroplast development affect ozone tolerance through the regulation of stomatal movement. Proc. Natl. Acad. Sci. U S A. 113 (15), 4218–4223. doi: 10.1073/pnas.1513093113 27035938 PMC4839443

[B136] Nanni A. V. Morse A. M. Newman J. R. B. Choquette N. E. Wedow J. M. Liu Z.. (2022). Variation in leaf transcriptome responses to elevated ozone corresponds with physiological sensitivity to ozone across maize inbred lines. Genetics 221, iyac080. doi: 10.1093/genetics/iyac080 35579358 PMC9339315

[B137] Navrot N. Rouhier N. Gelhaye E. Jacquot J. (2007). Reactive oxygen species generation and antioxidant systems in plant mitochondria. Physiol. Plant. 129, 185–195. doi: 10.1111/j.1399-3054.2006.00777.x

[B138] Neill S. Bright J. Desikan R. Hancock J. Harrison J. Wilson I. (2007). Nitric oxide evolution and perception. J. Exp. Bot. 59 (1), 25–35. doi: 10.1093/jxb/erm218 17975211

[B139] Neill S. J. Desikan R. Hancock J. T. (2003). Nitric oxide signalling in plants. New Phytol. 159 (1), 11–35. doi: 10.1046/j.1469-8137.2003.00804.x 33873677

[B140] Niles J. C. Wishnok J. S. Tannenbaum S. R. (2001). A novel nitroimidazole compound formed during the reaction of peroxynitrite with 2’,3′,5′-tri-O-acetyl-guanosine. J. Am. Chem. Soc 123, 12147–12151. doi: 10.1021/ja004296k 11734012

[B141] Nishida M. Kumagai Y. Ihara H. Fujii S. Motohashi H. Akaike T. (2016). Redox signaling regulated by electrophiles and reactive sulfur species. J. Clin. Biochem. Nutr. 58, 91–98. doi: 10.3164/jcbn.15-111 27013774 PMC4788399

[B142] Ohshima H. Sawa T. Akaike T. (2006). 8-nitroguanine, a product of nitrative DNA damage caused by reactive nitrogen species: formation, occurrence, and implications in inflammation and carcinogenesis. Antioxid. Redox Signal. 8, 1033–1045. doi: 10.1089/ars.2006.8.1033 16771693

[B143] Orozco-Cárdenas M. L. Ryan C. A. (2002). Nitric oxide negatively modulates wound signaling in tomato plants. Plant Physiol. 130 (1), 487–493. doi: 10.1104/pp.008375 12226527 PMC166580

[B144] Overmyer K. Brosché M. Pellinen R. Kuittinen T. Tuominen H. Ahlfors R.. (2005). Ozone-induced programmed cell death in the arabidopsis radical-induced cell death1 mutant. Plant Physiol. 137, 1092–1104. doi: 10.1104/pp.104.055681 15728341 PMC1065409

[B145] Padilla M. N. Mata-Pérez C. Melguizo M. Barroso J. B. (2017). *In vitro* nitro-fatty acid release from cys-NO_2_-fatty acid adducts under nitro-oxidative conditions. Nitric. Oxide 68, 14–22. doi: 10.1016/j.niox.2016.12.009 28030780

[B146] Pande A. Mun B. G. Rahim W. Khan M. Lee D. S. Lee G. M.. (2022). Phytohormonal regulation through protein s-nitrosylation under stress. Front. Plant Sci. 13. doi: 10.3389/FPLS.2022.865542 PMC8988057 35401598

[B147] París R. Lamattina L. Casalongué C. A. (2007). Nitric oxide promotes the wound-healing response of potato leaflets. Plant Physiol. Biochem. 45 (1), 80–86. doi: 10.1016/j.plaphy.2006.12.001 17280836

[B148] Perazzolli M. Dominici P. Romero-Puertas M. C. Zago E. Zeier J. Sonoda M.. (2004). Arabidopsis nonsymbiotic hemoglobin AHb1 modulates nitric oxide bioactivity. Plant Cell. 16, 2785–2794. doi: 10.1105/tpc.104.025379 15367716 PMC520971

[B149] Pinlaor S. Yongvanit P. Hiraku Y. Ma N. Semba R. Oikawa S.. (2003). 8-nitroguanine formation in the liver of hamsters infected with opisthorchis viverrini. Biochem. Biophys. Res. Commun. 309, 567–571. doi: 10.1016/j.bbrc.2003.08.039 12963027

[B150] Pucciariello C. Parlanti S. Banti V. Novi G. Perata P. (2012). Reactive oxygen species-driven transcription in arabidopsis under oxygen Deprivation1[W]. Plant Physiol. 159, 184–196. doi: 10.1104/pp.111.191122 22415514 PMC3375960

[B151] Puckette M. C. Tang Y. Mahalingam R. (2008). Transcriptomic changes induced by acute ozone in resistant and sensitive medicago truncatula accessions. BMC Plant Biol. 8, 46. doi: 10.1186/1471-2229-8-46 18433496 PMC2395263

[B152] Puyaubert J. Baudouin E. (2014a). New clues for a cold case: nitric oxide response to low temperature. Plant Cell Environ. 37 (12), 2623–2630. doi: 10.1111/pce.12329 24720833

[B153] Puyaubert J. Fares A. Rézé N. Peltier J.-B. Baudouin E. (2014b). Identification of endogenously s-nitrosylated proteins in arabidopsis plantlets: effect of cold stress on cysteine nitrosylation level. Plant Sci. 215-216, 150–156. doi: 10.1016/j.plantsci.2013.10.014 24388526

[B154] Qi Q. Yanyan D. Yuanlin L. Kunzhi L. Huini X. Xudong S. (2020). Overexpression of SlMDHAR in transgenic tobacco increased salt stress tolerance involving s-nitrosylation regulation. Plant Sci. 299, 110609. doi: 10.1016/j.plantsci.2020.110609 32900447

[B155] Radi R. (2004). Nitric oxide, oxidants and protein tyrosine nitration. Proc. Natl. Acad. Sci. U.S.A. 101, 4003–4008. doi: 10.1073/pnas.0307446101 15020765 PMC384685

[B156] Radi R. (2012). Protein tyrosine nitration: biochemical mechanisms and structural basis of functional effects. Acc. Chem. Res. 46, 550–559. doi: 10.1021/ar300234c 23157446 PMC3577981

[B157] Rajhi I. Yamauchi T. Takahashi H. Nishiuchi S. Shiono K. Watanabe R.. (2011). Identification of genes expressed in maize root cortical cells during lysigenous aerenchyma formation using laser microdissection and microarray analyses. New Phytol. 190, 351–368. doi: 10.1111/j.1469-8137.2010.03535.x 21091694

[B158] Rascio N. Navari-Izzo F. (2011). Heavy metal hyperaccumulating plants: How and why do they do it? and what makes them so interesting? Plant Sci. 180, 169–181. doi: 10.1016/J.PLANTSCI.2010.08.016 21421358

[B159] Raychaudhuri S. Pramanick P. Talukder P. Basak A. (2021). “Polyamines, metallothioneins, and phytochelatins–natural defense of plants to mitigate heavy metals,” in Studies in natural products chemistry (Elsevier), 227–261. doi: 10.1016/B978-0-12-819487-4.00006-9

[B160] Rehrig E. M. Appel H. M. Jones A. D. Schultz J. C. (2014). Roles for jasmonate- and ethylene-induced transcription factors in the ability of arabidopsis to respond differentially to damage caused by two insect herbivores. Front. Plant Sci. 5. doi: 10.3389/fpls.2014.00407 PMC4137388 25191332

[B161] Reymond P. Farmer E. E. (1998). Jasmonate and salicylate as global signals for defense gene expression. Curr. Opin. Plant Biol. 1 (5), 404–411. doi: 10.1016/s1369-5266(98)80264-1 10066616

[B162] Reymond P. Weber H. Damond M. Farmer E. E. (2000). Differential gene expression in response to mechanical wounding and insect feeding in arabidopsis. Plant Cell 12 (5), 707–719. doi: 10.1105/tpc.12.5.707 10810145 PMC139922

[B163] Romero-Puertas M. C. Rodríguez-Serrano M. Sandalio L. M. (2013). Protein s-nitrosylation in plants under abiotic stress: an overview. Front. Plant Sci. 4. doi: 10.3389/FPLS.2013.00373 PMC3778396 24065977

[B164] Rossi S. Burgess P. Jespersen D. Huang B. (2017). Heat-induced leaf senescence associated with chlorophyll metabolism in bentgrass lines differing in heat tolerance. Crop Sci. 57, 169–178. doi: 10.2135/cropsci2016.06.0542

[B165] Royo B. Moran J. F. Ratcliffe R. G. Gupta K. J. (2015). Nitric oxide induces the alternative oxidase pathway in arabidopsis seedlings deprived of inorganic phosphate. J. Exp. Bot. 66, 6273–6280. doi: 10.1093/jxb/erv338 26163703 PMC4588884

[B166] Rubio M. C. Calvo-Begueria L. Díaz-Mendoza M. Elhiti M. Moore M. Matamoros M. A.. (2019). Phytoglobins in the nuclei, cytoplasm and chloroplasts modulate nitric oxide signaling and interact with abscisic acid. Plant J. 100, 38–54. doi: 10.1111/tpj.14422 31148289

[B167] Sainz M. Calvo-Begueria L. Pérez-Rontomé C. Wienkoop S. Abián J. Staudinger C.. (2015). Leghemoglobin is nitrated in functional legume nodules in a tyrosine residue within the heme cavity by a nitrite/peroxide-dependent mechanism. Plant J. 81, 723–735. doi: 10.1111/tpj.12762 25603991 PMC4346251

[B168] Sánchez-McSweeney A. González-Gordo S. Aranda-Sicilia M. N. Rodríguez-Rosales M. P. Venema K. Palma J. M.. (2021). Loss of function of the chloroplast membrane K /H antiporters AtKEA1 and AtKEA2 alters the ROS and NO metabolism but promotes drought stress resilience. Plant Physiol. Biochem. 160, 106–119. doi: 10.1016/j.plaphy.2021.01.010 33485149

[B169] Sánchez-Vicente I. Lorenzo O. (2021). Nitric oxide regulation of temperature acclimation: a molecular genetic perspective. J. Exp. Bot. 72 (16), 5789–5794. doi: 10.1093/jxb/erab049 33544147 PMC8355752

[B170] Sasidharan R. Bailey-Serres J. Ashikari M. Atwell B. J. Colmer T. D. Fagerstedt K.. (2017). Community recommendations on terminology and procedures used in flooding and low oxygen stress research. New Phytol. 214, 1403–1407. doi: 10.1111/nph.14519 28277605

[B171] Sasidharan R. Hartman S. Liu Z. Martopawiro S. Sajeev N. van Veen H.. (2018). Signal dynamics and interactions during flooding Stress1[OPEN]. Plant Physiol. 176, 1106–1117. doi: 10.1104/pp.17.01232 29097391 PMC5813540

[B172] Sasidharan R. Schippers J. H. M. Schmidt R. R. (2021). Redox and low-oxygen stress: signal integration and interplay. Plant Physiol. 186, 66–78. doi: 10.1093/plphys/kiaa081 33793937 PMC8154046

[B173] Sawa T. Zaki M. H. Okamoto T. Akuta T. Tokutomi Y. Kim-Mitsuyama S.. (2007). Protein s-guanylation by the biological signal 8-nitroguanosine 3′,5′-cyclic monophosphate. Nat. Chem. Biol. 3, 727–735. doi: 10.1038/nchembio.2007.33 17906641

[B174] Saxena I. Shekhawat G. S. (2013). Nitric oxide (NO) in alleviation of heavy metal induced phytotoxicity and its role in protein nitration. Nitric. Oxide 32, 13–20. doi: 10.1016/J.NIOX.2013.03.004 23545403

[B175] Schopfer F. J. Cipollina C. Freeman B. A. (2011). Formation and signaling actions of electrophilic lipids. Chem. Rev. 111 (10), 5997–6021. doi: 10.1021/cr200131e 21928855 PMC3294277

[B176] Sehgal A. Sita K. Siddique K. H. M. Kumar R. Bhogireddy S. Varshney R. K.. (2018). Drought or/and heat-stress effects on seed filling in food crops: Impacts on functional biochemistry, seed yields, and nutritional quality. Front. Plant Sci. 9. doi: 10.3389/fpls.2018.01705 PMC6277783 30542357

[B177] Sehrawat A. Gupta R. Deswal R. (2013). Nitric oxide-cold stress signalling cross-talk, evolution of a novel regulatory mechanism. Proteomics 13, 1816–1835. doi: 10.1002/pmic.201200445 23580434

[B178] Sehrawat A. Sougrakpam Y. Deswal R. (2019). Cold modulated nuclear s-nitrosoproteome analysis indicates redox modulation of novel brassicaceae specific, myrosinase and napin in brassica juncea. Environ. Exp. Bot. 161, 312–333. doi: 10.1016/j.envexpbot.2018.10.010

[B179] Selinski J. Hartmann A. Kordes A. Deckers-Hebestreit G. Whelan J. Scheibe R. (2017). Analysis of posttranslational activation of alternative oxidase isoforms. Plant Physiol. 174, 2113–2127. doi: 10.1104/pp.17.00681 28596420 PMC5543971

[B180] Selinski J. Scheibe R. Day D. A. Whelan J. (2018). Alternative oxidase is positive for plant performance. Trends Plant Sci. 23 (7), 588–597. doi: 10.1016/j.tplants.2018.03.012 29665989

[B181] Serpa V. Vernal J. Lamattina L. Grotewold E. Cassia R. Terenzi H. (2007). Inhibition of AtMYB2 DNA-binding by nitric oxide involves cysteine s-nitrosylation. Biochem. Biophys. Res. Commun. 361, 1048–1053. doi: 10.1016/j.bbrc.2007.07.133 17686455

[B182] Shi Y. Ke X. Yang X. Liu Y. Hou X. (2022). Plants response to light stress. J. Genet. Genomics 49 (8), 735–747. doi: 10.1016/j.jgg.2022.04.017 35580763

[B183] Shi H. T. Li R. J. Cai W. Liu W. Wang C. L. Lu Y. T. (2012). Increasing nitric oxide content in arabidopsis thaliana by expressing rat neuronal nitric oxide synthase resulted in enhanced stress tolerance. Plant Cell Physiol. 53 (2), 344–357. doi: 10.1093/pcp/pcr181 22186181

[B184] Si T. Wang X. Wu L. Zhao C. Zhang L. Huang M.. (2017). Nitric oxide and hydrogen peroxide mediate wounding-induced freezing tolerance through modifications in photosystem and antioxidant system in wheat. Front. Plant Sci. 8. doi: 10.3389/fpls.2017.01284 PMC5515872 28769973

[B185] Sierla M. Hõrak H. Overmyer K. Waszczak C. Yarmolinsky D. Maierhofer T.. (2018). The receptor-like pseudokinase GHR1 is required for stomatal closure. Plant Cell 30, 2813–2837. doi: 10.1105/tpc.18.00441 30361234 PMC6305979

[B186] Skubacz A. Daszkowska-Golec A. Szarejko I. (2016). The role and regulation of ABI5 (ABA-insensitive 5) in plant development, abiotic stress responses and phytohormone crosstalk. Front. Plant Sci. 7. doi: 10.3389/fpls.2016.01884 PMC5159420 28018412

[B187] Spoel S. H. Van Ooijen G. (2014). Circadian redox signaling in plant immunity and abiotic stress. Antioxid. Redox Signal 20 (18), 3024–3039. doi: 10.1089/ars.2013.5530 23941583 PMC4038994

[B188] Srivastava M. Ma L. Q. Singh N. Singh S. (2005). Antioxidant responses of hyper-accumulator and sensitive fern species to arsenic. J. Exp. Bot. 56, 1335–1342. doi: 10.1093/JXB/ERI134 15781440

[B189] Suzuki N. Mittler R. (2012). Reactive oxygen species-dependent wound responses in animals and plants. Free Radical Biol. Med. 53 (12), 2269–2276. doi: 10.1016/j.freeradbiomed.2012.10.538 23085520

[B190] Swamy P. M. Smith B. N. (1999). Role of abscisic acid in plant stress tolerance. Curr. Sci. 76, 1220–1227.

[B191] Tai A. P. K. Sadiq M. Pang J. Y. S. Yung David H. Y. Feng Z. (2021). Impacts of surface ozone pollution on global crop yields: comparing different ozone exposure metrics and incorporating co-effects of CO2. Front. Sustain. Food Syst. 29. doi: 10.3389/fsufs.2021.534616

[B192] Tanou G. Ziogas V. Belghazi M. Christou A. Filippou P. Job D.. (2014). Polyamines reprogram oxidative and nitrosative status and the proteome of citrus plants exposed to salinity stress. Plant Cell Environ. 37, 864–885. doi: 10.1111/pce.12204 24112028

[B193] Toh S. Imamura A. Watanabe A. Nakabayashi K. Okamoto M. Jikumaru Y.. (2008). High temperature-induced abscisic acid biosynthesis and its role in the inhibition of gibberellin action in arabidopsis seeds. Plant Physiol. 146, 1368–1385. doi: 10.1104/pp.107.113738 18162586 PMC2259091

[B194] Tossi V. Lamattina L. Jenkins G. I. Cassia R. O. (2014). ). ultraviolet-b-induced stomatal closure in arabidopsis is regulated by the UV RESISTANCE LOCUS8 photoreceptor in a nitric oxide-dependent mechanism. Plant Physiol. 164 (4), 2220–2230. doi: 10.1104/pp.113.231753 24586043 PMC3982774

[B195] Trapet P. Kulik A. Lamotte O. Jeandroz S. Bourque S. Nicolas-Frances V.. (2015). NO signaling in plant immunity: A tale of messengers. Phytochemistry 112, 72–79. doi: 10.1016/j.phytochem.2014.03.015 24713571

[B196] Turkan I. (2018). ROS and RNS: key signalling molecules in plants. J. Exp. Bot. 69, 3313–3315. doi: 10.1093/jxb/ery198 29931350 PMC6009602

[B197] Valderrama R. Corpas F. J. Carreras A. Fernández-Ocaña A. Chaki M. Luque F.. (2007). Nitrosative stress in plants. FEBS Lett. 581, 453–461. doi: 10.1016/j.febslet.2007.01.006 17240373

[B198] Vanhaelewyn L. Prinsen E. van der Straeten D. Vandenbussche F. (2016). Hormone-controlled UV-b responses in plants. J. Exp. Bot. 67 (15), 4469–4482. doi: 10.1093/jxb/erw261 27401912

[B199] Vanzo E. Ghirardo A. Merl-Pham J. Lindemayr C. Heller W. Hauck S. M.. (2014). S-nitroso-proteome in poplar leaves in response to acute ozone stress. PloS One 9 (9), e106886. doi: 10.1371/journal.pone.0106886 25192423 PMC4156402

[B200] Vishwakarma A. Kumari A. Mur L. A. J. Gupta K. J. (2018). A discrete role for alternative oxidase under hypoxia to increase nitric oxide and drive energy production. Free Radic. Biol. Med. 122, 40–51. doi: 10.1016/j.freeradbiomed.2018.03.045 29604396

[B201] Vistoli G. De Maddis D. Cipak A. Zarkovic N. Carini M. Aldini G. (2013). Advanced glycoxidation and lipoxidation end products (AGEs and ALEs): an overview of their mechanisms of formation. Free Radic. Res. 47 (Suppl 1), 3–27. doi: 10.3109/10715762.2013.815348 23767955

[B202] Vollár M. Feigl G. Oláh D. Horváth A. Molnár Á. Kúsz N.. (2020). Nitro-oleic acid in seeds and differently developed seedlings of brassica napus l. Plants (basel Switzerland) 9 (3), 406. doi: 10.3390/plants9030406 32214020 PMC7154869

[B203] Wang Q. L. Chen J. H. He N. Y. Guo F. Q. (2018). Metabolic reprogramming in chloroplasts under heat stress in plants. Int. J. Mol. Sci. 19, 849. doi: 10.3390/ijms19030849 29538307 PMC5877710

[B204] Wang P. Du Y. Hou Y. Zhao Y. Hsu C. Yuan F.. (2015). Nitric oxide negatively regulates abscisic acid signaling in guard cells by s-nitrosylation of OST1. Proc. Natl. Acad. Sci. 112, 613–618. doi: 10.1073/pnas.1423481112 25550508 PMC4299189

[B205] Wang C. Wei L. Zhang J. Hu D. Gao R. Liu Y.. (2022). Nitric oxide enhances salt tolerance in tomato seedlings by regulating endogenous s-nitrosylation levels. J. Plant Growth Regul. 42, 275–293. doi: 10.1007/s00344-021-10546-5

[B206] Wany A. Kumari A. Gupta K. J. (2017). Nitric oxide is essential for the development of aerenchyma in wheat roots under hypoxic stress. Plant Cell Environ. 40 (12), 3002–3017. doi: 10.1111/pce.13061 28857271

[B207] Wasternack C. Song S. (2017). Jasmonates: biosynthesis, metabolism, and signaling by proteins activating and repressing transcription. J. Exp. Bot. 68 (6), 1303–1321. doi: 10.1093/jxb/erw443 27940470

[B208] Waszczak C. Carmody M. Kangasja¨rvi J. (2018). Reactive oxygen species in plant signaling. Annu. Rev. Plant Biol. 69, 209–236. doi: 10.1146/annurev-arplant-042817-040322 29489394

[B209] Wei L. Zhang J. Wei S. Hu D. Liu Y. Feng L.. (2022). Nitric oxide enhanced salt stress tolerance in tomato seedlings, involving phytohormone equilibrium and photosynthesis. Int. J. Mol. Sci. 23, 4539. doi: 10.3390/ijms23094539 35562930 PMC9102644

[B210] Weits D. A. van Dongen J. T. Licausi F. (2021). Molecular oxygen as a signaling component in plant development. New Phytol. 229, 24–35. doi: 10.1111/nph.16424 31943217

[B211] Wrzaczek M. Brosché M. Salojärvi J. Kangasjärvi S. Idänheimo N. Mersmann S.. (2010). Transcriptional regulation of the CRK/DUF26 group of receptor-like protein kinases by ozone and plant hormones in arabidopsis. BMC Plant Biol. 10, 95. doi: 10.1186/1471-2229-10-95 20500828 PMC3095361

[B212] Wu R. Agathokleous E. Yung D. H. Y. Tai A. P. K. Shang B. Feng Z. (2022). Joint impacts of ozone pollution and climate change on yields of Chinese winter wheat. Atmospheric pollut. Research 13, 101509. doi: 10.1016/j.apr.2022.101509

[B213] Wu Q. Su N. Zhang X. Liu Y. Cui J. Liang Y. (2016). Hydrogen peroxide, nitric oxide and UV RESISTANCE LOCUS8 interact to mediate UV-b-induced anthocyanin biosynthesis in radish sprouts. Sci. Rep. 6, 29164. doi: 10.1038/srep29164 27404993 PMC4941517

[B214] Xu E. Vaahtera L. H~orak H. Hincha D. K. Heyer A. G. Brosché M. (2015). Quantitative trait loci mapping and transcriptome análisis reveal candidate genes regulating the response to ozone in arabidopsis thaliana. Plant Cell Environ. 38, 1418–1433. doi: 10.1111/pce.12499 25496229

[B215] Xu J. Wang W. Yin H. Liu X. Sun H. Mi Q. (2010). Exogenous nitric oxide improves antioxidative capacity and reduces auxin degradation in roots of medicago truncatula seedlings under cadmium stress. Plant Soil 326, 321–330. doi: 10.1007/s11104-009-0011-4

[B216] Xu M. Zhu Y. Dong J. Jin H. Sun L. Wang Z.. (2012). Ozone induces flavonol production of ginkgo biloba cells dependently on nitrate reductase mediated nitric oxide signaling. Envir. Exp. Bot. 75, 114–119. doi: 10.1016/j.envexpbot.2011.09.005

[B217] Xuan Y. Zhou S. Wang L. Cheng Y. Zhao L. (2010). Nitric oxide functions as a signal and acts upstream of AtCaM3 in thermotolerance in arabidopsis seedlings. Plant Physiol. 153, 1895–1906. doi: 10.1104/pp.110.160424 20576787 PMC2923878

[B218] Yamauchi T. Watanabe K. Fukazawa A. Mori H. Abe F. Kawaguchi K.. (2014). Ethylene and reactive oxygen species are involved in root aerenchyma formation and adaptation of wheat seedlings to oxygen-deficient conditions. J. Exp. Bot. 65, 261–273. doi: 10.1093/jxb/ert371 24253196 PMC3883296

[B219] Yamauchi T. Yoshioka M. Fukazawa A. Mori H. Nishizawa N. K. Tsutsumi N.. (2017). An NADPH oxidase RBOH functions in rice roots during lysigenous aerenchyma formation under oxygen-deficient conditions. Plant Cell 29, 775–790. doi: 10.1105/tpc.16.00976 28351990 PMC5435434

[B220] Yermilov V. Rubio J. Becchi M. Friesen M. D. Pignatelli B. Ohshima H. (1995a). Formation of 8-nitroguanine by the reaction of guanine with peroxynitrite *in vitro* . Carcinogenesis 16, 2045–2050. doi: 10.1093/carcin/16.9.2045 7554052

[B221] Yermilov V. Rubio J. Ohshima H. (1995b). Formation of 8-nitroguanine in DNA treated with peroxynitrite *in vitro* and its rapid removal from DNA by depurination. FEBS Lett. 376, 207–210. doi: 10.1016/0014-5793(95)01281-6 7498543

[B222] Yeung E. van Veen H. Vashisht D. Sobral Paiva A. L. Hummel M. Rankenberg T.. (2018). A stress recovery signaling network for enhanced flooding tolerance in arabidopsis thaliana. Proc. Natl. Acad. Sci. U. S. A. 115, E6085–E6094. doi: 10.1073/pnas.1803841115 29891679 PMC6042063

[B223] Yin X. Hu Y. Meng L. Zhang X. Liu H. Wang L.. (2021). Effects of exogenous nitric oxide on wild barley (Hordeum brevisubulatum) under salt stress. Biotechnol. Biotechnol. Equip. 1, 2005–2016. doi: 10.1080/13102818.2022.2041096

[B224] Yin L. Mano J. Wang S. Tsuji W. Tanaka K. (2010). The involvement of lipid peroxide-derived aldehydes in aluminum toxicity of tobacco roots plant physiol. Plant. Physiol. 152, 1406–1417. doi: 10.1104/pp.109.151449 20023145 PMC2832258

[B225] Ying S. Yang W. Li P. Hu Y. Lu S. Zhou Y.. (2022). Phytochrome b enhances seed germination tolerance to high temperature by reducing s-nitrosylation of HFR1. EMBO Rep. 23, e54371. doi: 10.15252/embr.202154371 36062942 PMC9535752

[B226] Yu M. Lamattina L. Spoel S. H. Loake G. J. (2014). Nitric oxide function in plant biology: A redox cue in deconvolution. New Phytol. 202, 1142–1156. doi: 10.1111/nph.12739 24611485

[B227] Yun B.-W. Feechan A. Yin M. Saidi N. B. B. Le Bihan T. Yu M.. (2011). S-nitrosylation of NADPH oxidase regulates cell death in plant immunity. Nature 478, 264–268. doi: 10.1038/nature10427 21964330

[B228] Zandi P. Schnug E. (2022). Reactive oxygen species, antioxidant responses and implications from a microbial modulation perspective. Biol. (Basel) 11, 155–185. doi: 10.3390/BIOLOGY11020155 PMC8869449 35205022

[B229] Zhan N. Wang C. Chen L. Yang H. Feng J. Gong X.. (2018). S-nitrosylation targets GSNO reductase for selective autophagy during hypoxia responses in plants. Mol. Cell 71, 142–154. doi: 10.1016/j.molcel.2018.05.024 30008318

[B230] Zhang M. An L. Feng H. Chen T. Chen K. Liu Y.. (2003). The cascade mechanisms of nitric oxide as a second messenger of ultraviolet b in inhibiting mesocotyl elongations. Photochem. Photobiol. 77 (2), 219–225. doi: 10.1562/0031-8655(2003)077<0219:tcmono>2.0.co;2 12785062

[B231] Zhang J. Wang Y. Mao Z. Liu W. Ding L. Zhang X.. (2022). Transcription factor McWRKY71 induced by ozone stress regulates anthocyanin and proanthocyanidin biosynthesis in malus crabapple. Ecotoxicol. Environ. Saf. 232, 113274. doi: 10.1016/j.ecoenv.2022.113274 35124421

[B232] Zhao H. Qian R. Liang X. Ou Y. Sun C. Lin X. (2022). Indium induces nitro-oxidative stress in roots of wheat (Triticum aestivum). J. Hazard. Mater. 428, 128260. doi: 10.1016/J.JHAZMAT.2022.128260 35038664

[B233] Zitka O. Krystofova O. Hynek D. Sobrova P. Kaiser J. Sochor J.. (2013). Metal transporters in plants (Berlin Heidelberg: Springer-Verlag), 19–41. doi: 10.1007/978-3-642-38469-1_2

